# Thermal Phase Behavior of PNIPAM-Based Hydrogels: Different Scenarios and Implications in Injectable Hydrogel Design

**DOI:** 10.3390/gels12100930

**Published:** 2026-10-15

**Authors:** Svetlana L. Kotova, Valeriya S. Kukanova, Sergei V. Kostjuk, Yuri M. Efremov, Yury A. Rochev, Anastasia A. Frolova, Peter S. Timashev

**Affiliations:** 1 Institute for Regenerative Medicine, Sechenov University, 8-2 Trubetskaya St., 119991 Moscow, Russia; kukanova_v_s@staff.sechenov.ru (V.S.K.); efremov_yu_m@staff.sechenov.ru (Y.M.E.); frolova_a_a_2@staff.sechenov.ru (A.A.F.); timashev_p_s@staff.sechenov.ru (P.S.T.); 2 Equipe Chimie des Polymeres, Institut Parisien de Chimie Moleculaire, Sorbonne Universite, Centre national de la recherche scientifique, 4 Place Jussieu, 75252 Cedex 05 Paris, France; sergei.kostjuk@sorbonne-universite.fr; 3 School of Natural Sciences, Physics, University of Galway, H91 CF50 Galway, Ireland; yury.rochev@nuigalway.ie; 4 CÚRAM, Research Ireland Centre for Medical Devices, University of Galway, H91 W2TY Galway, Ireland; 5 Department of Biological Chemistry, Sechenov University, 8-2 Trubetskaya St., 119991 Moscow, Russia

**Keywords:** poly(N-isopropylacrylamide), smart materials, thermoresponsive polymers, injectable hydrogels, coil-to-globule transition, gelation

## Abstract

Poly(N-isopropylacrylamide) (PNIPAM)-based hydrogels are “smart” materials of great interest and demand, primarily in a variety of biomedical applications, due to their LCST close to physiological temperature. The basic biomedical applications of PNIPAM-based injectable hydrogels include controlled drug delivery, tissue engineering, wound healing, and cancer therapy. This review summarizes the thermal phase behavior of PNIPAM-based systems under different conditions, including different scenarios of physical gelation in polymer solutions and volume phase transitions in chemically crosslinked hydrogels. The thermodynamics of the coil-to-globule phase transition, its relation to gel formation, and mechanisms underlying the phase transitions in PNIPAM-based hydrogels are discussed, as well as external parameters which may affect these processes. Different types of phase diagrams observed for PNIPAM-based systems are considered, with particular attention paid to the cases in which gelation occurs separately from the coil-to-globule transition. The rational design of injectable platforms should be based upon a thorough understanding of the PNIPAM-based system’s phase behavior to achieve controllable and reproducible properties.

## 1. Introduction

### 1.1. Thermoresponsive Hydrogels

Stimuli-responsive, or “smart” hydrogels attract great interest due to their unique phase behavior, primarily for a variety of biomedical applications, but also for emerging applications in other fields. Stimuli-responsive hydrogels exhibit a dramatic change in their swelling, mechanical properties and shape upon a shift in the environmental conditions, such as temperature, pH, ionic strength, light wavelength, electric and magnetic fields. Thermoresponsive polymers are among the most extensively studied smart materials, since their phase behavior may be governed by the body temperature, which determines their great potential in medical uses. The phase diagrams of such systems are characterized by a binodal which divides the regions of one-phase and two-phase states, with an upper critical solution temperature (UCST), a lower critical solution temperature (LCST), or both. The majority of prospective thermoresponsive hydrogels are based on LCST-type polymers, due to the higher feasibility of such approaches. The recent advances in these approaches are collected in a large number of reviews which have appeared in recent years (see, e.g., [1,2,3,4,5,6,7,8,9,10]).

There are many classes of thermoresponsive polymers forming hydrogels, including poloxamers, poly(N-vinylcaprolactam), poly(N-ethyloxazoline), poly(methyl vinyl ether) and acrylamide-based copolymers [1,7,11]. However, various derivatives of poly-N-isopropylacrylamide (PNIPAM) have attracted the most attention due to their transition temperature close to the body temperature. PNIPAM has an LCST of about 32 °C, and addition of different hydrophilic and hydrophobic units may shift it higher or lower, in accordance with the target application. When heated to the LCST, aqueous solutions of PNIPAM-based polymers demix and form two phases, while, starting from a certain concentration, they may form physical gels. At the same time, when crosslinked, swollen hydrogels release the bound water and undergo a collapse at the transition temperature (volume phase transition temperature, VPTT). Polymer solutions forming a physical gel upon heating are termed “thermoreversible gels” or “thermogelling materials”, their mechanisms of gelation depending on the design of their macromolecules [10].

The key feature of a hydrogel’s structure, whether it is a physical or a chemical gel, is a 3D network of polymer chains which absorb and retain a high amount of water, while sustaining its integrity and mechanical characteristics [6,12]. The water-filled regions of the network may be varied in their sizes to provide transport of various molecules or support whole cells. Such a specific structure and properties are due to the presence of hydrophilic functional groups, such as -OH, -SO_3_H, -COOH, -NH_2_ and -CONH_2_, in the macromolecules comprising a hydrogel, which further promote the hydrogel interactions with live tissues [12].

Physical hydrogels are held together by non-covalent bonds such as hydrogen bonds, hydrophobic interactions, ionic interactions, and chain entanglement [6,12]. Physical hydrogels undergo reversible gelation and may be returned to the liquid state upon lowering the temperature. They can also be used as self-healing hydrogels [6]. Chemical crosslinking includes the formation of chemical bridges using various crosslinkers (bis-acrylamide, glutaraldehyde, elastin-like polypeptides, alginate, photosensitive agents, enzymatic crosslinkers), and provides a reproducible and stable 3D network [5,6,12]. Chemically crosslinked thermoresponsive hydrogels range in their sizes from bulk materials at the macroscale to microgels (~100 nm to microns) and nanogels at the nanoscale [5].

The sol–gel transition is monitored using various techniques, primarily by rheological methods, with the frequently used simplified approach to defining the gelation temperature as the point at which the elastic modulus, G′, starts to exceed the viscous modulus G′′ [11].

### 1.2. Applications of Thermoresponsive Hydrogels

The basic applications of thermoresponsive hydrogels are associated with biomedical engineering, primarily, with novel controlled-release drug-delivery systems [1,2].

The review by Throat and Bhattacharya [5] extensively discusses how PNIPAM-based systems are used in studies on cancer treatment. In particular, microgels and nanogels, as well as conjugates have appeared efficient in the sustained anti-cancer target drug delivery. A new thermoresponsive hydrogel system for generation and isolation of cancer cell spheroids is also described. Of the latest experimental studies on anticancer drug delivery using PNIPAM-based hydrogels, one can refer to [13,14]. Narayana et al. mention the application of such systems in the photothermal/photodynamic therapy of cancer [6]. PNIPAM-based nanospheres in conjunction with optical coherence tomography showed promise in cancer-related imaging [5]. Other drug-delivery systems with PNIPAM-based hydrogels included those for ocular diseases, oral ulcers, skin diseases, Parkinson’s disease, and antibacterial agents [5,6,15].

As mentioned above, hydrogels may be designed to sustain whole cells, which determines their importance in biological tissue engineering, including scaffolds for cartilage regeneration, dentoalveolar tissue engineering, intervertebral disc regeneration, and bone regeneration [5,6,16].

The intrinsic porosity and capacity to retain large volumes of water determine the application of PNIPAM-based hydrogels in wound dressings, since they are able not only to deliver the required drug payload to the wound, but also to support healing and tissue regeneration, as well as to carry agents for wound monitoring [3,6].

The emerging medical and sport-related application of PNIPAM hydrogels is associated with various sensors, including wearable temperature sensors, strain sensors, sensors measuring pressure and pH, chemosensors, humidity sensors, and biosensors for glucose, hydrogen peroxide, proteins, DNA, drugs, etc. (Refs. [2,7,17]). Along with sensors, smart textiles are also developed with the use of such systems [2]. A novel branch of sensor development, implantable bioelectronics, exploiting soft hydrogels with biomimetic properties, shows great promise for neurotechnologies [18].

The non-medical applications of PNIPAM-based hydrogels have attracted growing attention in recent years and include energy-saving smart windows and curtains (Refs. [2,19,20,21]), solar-driven interfacial evaporation [2], actuators and soft robotics [2], preservation of fruit and vegetables [22], anti-counterfeiting labels and information encryption devices [23,24], and smart rechargeable zinc-ion batteries [25].

### 1.3. Injectable PNIPAM-Based Hydrogels

Drug and cell delivery using injectable thermoresponsive hydrogels appears an advantageous way offering minimally invasive surgical intervention, enhanced bioavailability, and lower costs as compared to conventional surgical interventions [12,25,26,27,28,29]. Depending on the clinical applications, several administration options are described for injectable hydrogels, among them subcutaneous, intramuscular, intratumoral, intra-articular and intraocular injections [28].

**Figure 1:**
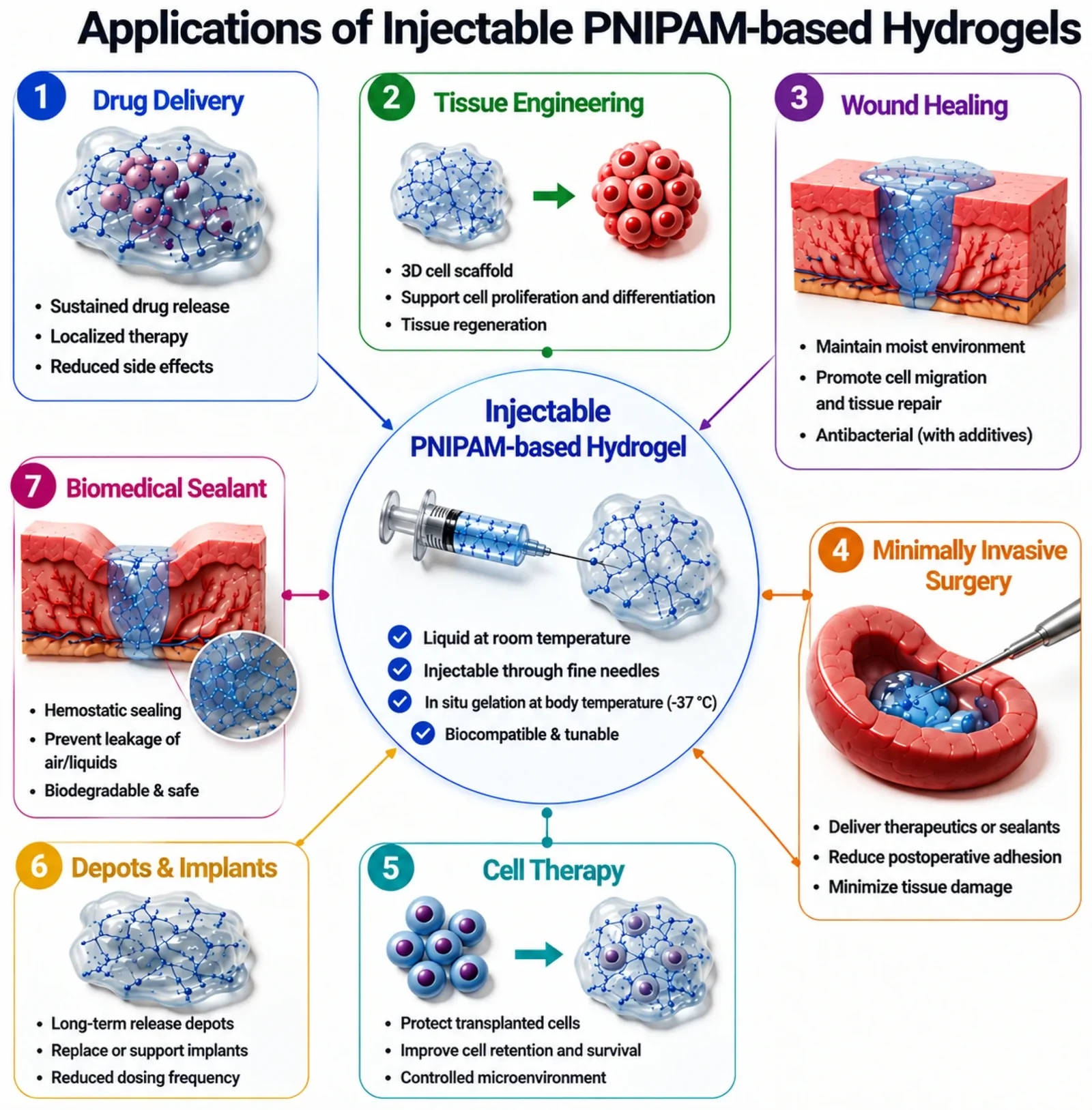
Basic potential applications of injectable hydrogels based on thermoresponsive PNIPAM derivatives.

The basic principle behind the injectable gel delivery consists in the hydrogel administration in the form of a solution via an injection, followed by the solution being heated naturally inside the body to the body temperature, which exceeds the temperature of gelation (phase transition). In drug delivery, the formed gel then releases the loaded cargo in a controlled manner, thus minimizing the systemic toxicity and related side effects. In tissue engineering applications, the cell-laden scaffold is injected into the damaged tissue as a viscous fluid without the need for surgery, providing the precise filling and sustained shape and robust mechanical properties afterwards, while maintaining the transport of needed biologically active molecules to the treated site [12,28]. Injectable hydrogels appear promising materials in cell therapy, as well [29]. The high potential of injectable thermoresponsive compositions is associated with the possibility of immediately connecting the synthetic material with the treated tissues, since the used polymeric systems are designed to mimic the natural extracellular matrix [28]. The basic applications of PNIPAM-based injectable hydrogels are displayed in Figure 1.

In cancer therapy, thermoresponsive hydrogel-based systems show promise in intratumoral delivery of chemotherapeutic drugs which otherwise may cause significant toxicity to healthy tissues [27]. An in situ formed gel functions as a prolonged release composition applied directly at the tumor site, thus allowing one to use relatively high drug doses. The recent review by Zhang et al. discusses the use of injectable hydrogels in the therapy of osteoarthritis [25].

Wound treatment also finds advantages in the use of injectable self-healing hydrogels [3]. Liquid polymers may take on any geometric shape of a wound, forming a sufficiently mechanically strong dressing after gelation, eliminating the need for suturing the wound. Moreover, they may provide controlled drug release during the wound healing process.

Interestingly, injectable PNIPAM-based hydrogels appeared instrumental in modeling certain medical conditions by inducing local stress on tissues upon their gelation [30].

In the design of injectable thermoresponsive hydrogels, the temperature of the coil-to-globule transition/gelation serves as the basic criterion for their performance. It should be noted, though, that the practical implication of thermoresponsiveness involves not a single temperature value, but rather an operational window: an injectable composition should be consistently liquid during handling and injection, but rapidly and reproducibly gel in the tissue after the injection, despite variations in the local temperature and composition. Besides the apparent importance of the gelation temperature, the viscous properties of the liquid phase should also be considered as the basic condition for injectability. The polymer concentration should be high enough to exceed the threshold concentration for gelation; at the same time, the higher the concentration, the higher the viscosity, and hence the poorer the injectability. In general, shear-thinning behavior is highly preferable, since, in this case, viscosity drops under shear force, which provides better extrusion of the solution [3]. The combination of the shear-thinning behavior with a high rate of network reassembly is needed to create a stable pool of the hydrogel at the target site [3].

There are four basic mechanisms described for drug release from hydrogels [26,31]—diffusion-controlled, erosion-controlled, chemically controlled, and swelling-controlled ones. The type of mechanism for a certain hydrogel is defined by the hydrogel mesh size and the cargo molecule size. Small molecules are transferred from the matrix via diffusion, which may be rather fast, while large molecules like proteins may diffuse much slower, proving the prolonged release. When the drug size exceeds the mesh size of the hydrogel, erosion- or swelling-controlled mechanisms are responsible for the drug release.

Biocompatibility and biodegradability are desirable properties for carrier hydrogels; thus, weak bonds are frequently used in the hydrogel design [26]. The degradation of PNIPAM in tissues results in the production of the monomer, cyclic imides, and diisopropylamine via chain scission; peptide fragments and products from crosslinkers may also be found; however, in general, PNIPAM shows low toxicity [6]. Surprisingly, little is known about the long-term fate of these polymers after the injection, since such investigations are rare. Bertrand et al. performed a study on the pharmacokinetics and biodistribution of PNIPAM copolymers [32] in rat tissues and showed that PNIPAM copolymers were accumulated preferentially in the liver, kidneys, spleen, and lungs, and were eliminated primarily by urinary excretion, with both processes depending on the copolymers’ molecular weight and chemical structure. They concluded that the preferable molecular weight of PNIPAM should be below 32 kDa and the transition temperature above 37 °C to guarantee the polymer elimination from the body.

Among other limitations of PNIPAM, insufficient mechanical strength of hydrogels and restricted ability to retain drugs leading to burst release are usually mentioned [4,6]. The burst release is related to the rapid collapse of the 3D network above the transition temperature with the expulsion of a great amount of water, which washes away small-sized cargo molecules. A more precise control over the hydrogel mesh size and the transition kinetics is desirable, then, when designing the delivery systems for a small-sized cargo. For the case of a large-sized cargo, such as proteins, another problem arises—PNIPAM is known for its skin-layer effect, i.e., a faster phase transition in the upper layer of a hydrogel, leading to the formation of a dense compacted surface layer. The latter serves as a barrier to penetration of large molecules and particles. Neat PNIPAM hydrogels are also prone to syneresis with fast gel shrinking, which further complicates sustained drug release. The gel heterogeneity is undesirable, as well, since it results in non-uniform diffusion of the cargo into the environment. A plethora of various approaches aimed at overcoming PNIPAM drawbacks and creating highly functional hydrogels are currently developed [4].

The above-mentioned limitations, along with the problems with the reproducible manufacturing, scalability and regulatory standards, as well as difficulties choosing an adequate animal model, represent challenges which slow the translation of the research results into clinical practice [28].

According to [28], 81 systems based on injectable hydrogels have been mentioned as products on the market and objects of clinical studies to date; however, no PNIPAM-based products have been included in this list. Nevertheless, a whole number of patents on PNIPAM-based smart hydrogel applications in biomedical fields can be found, mostly on cancer, wound healing, and ocular hydrogels [6].

The above analysis of the latest advances in the design of PNIPAM-based hydrogels shows that the primary focus of essentially all the reviews on this subject is the application of hydrogels. This subject is covered extensively, as compared to the physico-chemical aspects of their creation, which appear to be presented only superficially in the literature. Meanwhile, the thermal phase behavior of PNIPAM-based hydrogels forms the basis for all their applications, in particular, in the form of injectable hydrogels. Thus, the goal of this review is to cover the basic knowledge on the thermal transitions in PNIPAM-based hydrogels, including coil-to-globule transitions and gelation in solutions, volume phase transitions in chemically crosslinked hydrogels, discussing the external factors affecting these processes, with a special focus on different types of observed phase diagrams and different scenarios of phase transitions.

## 2. Thermodynamics of the Coil-to-Globule Transition in PNIPAM

### 2.1. Phase Diagram of PNIPAM

PNIPAM hydrogels belong to LCST-type hydrogels, including also hydrogels of such polymers as block-copolymers of poly(ethylene oxide) and poly(propylene oxide), poly(methyl vinyl ether), poly(2-hydroxypropyl acrylate), oxazoline-based polymers, etc. [2].

Polymers with an LCST form hydrated coils in aqueous solutions, with hydrophobic parts surrounded by ordered “water cages” [33]. Upon heating above the LCST, the polymer chains undergo massive dehydration, releasing a large amount of water, while hydrophobic interactions bring together polymer chains with the formation of compact globules. This coil-to-globule transition is responsible for the phase behavior of thermoresponsive polymers.

In terms of thermodynamics, this process is governed by the Gibbs free energy:

(1) ΔG_mix_ = ΔH_mix_ − TΔS_mix_

where ΔG_mix_, ΔH_mix_ and ΔS_mix_ are the Gibbs free energy, enthalpy and entropy of mixing, respectively.

The spontaneous dissolution takes place when ΔG_mix_ is negative, while ΔG_mix_ > 0 determines the polymer–water system demixing. Due to the formation of hydrogen bonds, the enthalpy of PNIPAM mixing with water is negative, favoring dissolution. Thus, ΔG_mix_ becomes positive when the term TΔS_mix_ exceeds the enthalpic gain. For a classical polymer solution, ΔS_mix_ is positive due to the possibility of a greater number of chain configurations [10]; however, in PNIPAM the chain hydration and formation of “water cages” around the hydrophobic moieties result in the opposite—the entropy gain upon demixing and releasing the bound water, so that ΔS_mix_ > 0 above the critical temperature. This balance between the hydration and dehydration processes leads to the existence of the LCST and makes the coil-to-globule transition in PNIPAM an entropy-driven process.

**Figure 2:**
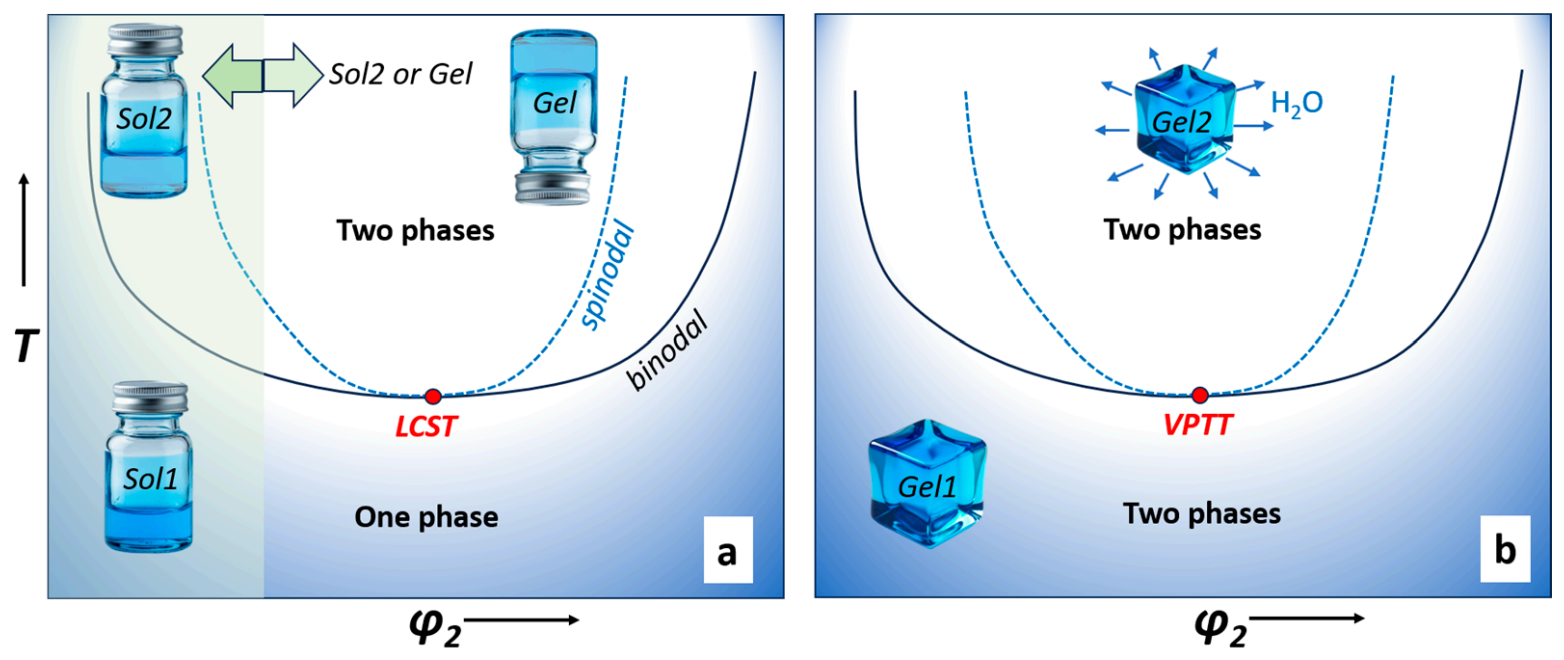
Phase diagrams of a PNIPAM-based polymer. (**a**)—polymer without cross-links. *Sol1*—transparent sol below the transition temperature, *Sol2*—turbid sol above the transition temperature, *Gel*—turbid gel above the transition temperature. The green band marks the region of concentrations too low for gel formation. At the higher concentrations, some PNIPAM-based (co)polymers are capable of forming physical gels, while other polymers precipitate. (**b**)—crosslinked polymer. *Gel1*—swollen transparent gel below the transition temperature. *Gel2*—turbid deswollen gel above the transition temperature. *φ*_2_ is the volume fraction of the polymer.

The presence of the minimum critical point leads to the characteristic U-shaped phase diagram of a PNIPAM–water system (Figure 2). In general, below the binodal the system exists as one phase in the form of a transparent sol of the polymer’s hydrated coils mixed with water molecules. Above the binodal, the system demixes: within the region between the binodal and spinodal, demixing proceeds via the nucleation and growth mechanism; above the spinodal, it proceeds via spinodal decomposition [34]. PNIPAM globules have a propensity to aggregate with the formation of mesoglobules [35], giving rise to turbidity upon demixing; thus, the term “cloud point” is frequently used interchangeably with “LCST”. However, it should be remembered that the cloud point curve, though lying close to the binodal, is not the thermodynamically correct phase diagram [34].

In regard to PNIPAM-based hydrogels, the coil-to-globule transition appears differently for free macromolecules and a crosslinked polymer. In the former case, the polymer–water mixture below the binodal represents a transparent sol. Upon heating to the transition temperature, it may form a turbid sol (or precipitate at a high enough concentration), or, starting from a certain concentration, some PNIPAM derivatives may undergo physical gelation, with the formation of a turbid gel (Figure 2a). In this case, a hydrogel is created via the coil-to-globule transition from a liquid. When the polymer is crosslinked, its macromolecules cannot form free coils which collapse into globules during the phase transition; thus, the system represents a hydrogel before and after the transition. Below the transition temperature, it is a highly swollen transparent hydrogel; upon heating above the transition temperature, it abruptly shrinks to a much lower volume, with the release of water, and becomes turbid (Figure 2b). Such a transition is termed a volume phase transition, and the minimum at the binodal is represented by a volume phase transition temperature (VPTT) vs. the LCST in an uncrosslinked polymer. Generally, VPTT does not necessarily coincide with LCST; however, PNIPAM is characterized by the almost universal transition temperature, independently of the polymer form.

It is of note that the latter property of PNIPAM causes the apparent overuse of the “LCST” term as applied to the phase transition in any PNIPAM form (powder, film, fiber, gel). Meanwhile, “LCST” assumes the transition in a solution, and a more general “coil-to-globule transition temperature” or a specialized term would be more appropriate for another polymer state. Table 1 summarizes the main critical temperature points used in the description of PNIPAM’s phase behavior.

According to the classical Flory–Huggins theory, the free Gibbs energy of mixing may be presented as

(2) Δ*G*_mix_ = *RT* (*n*_1_ln *φ*_1_ + *n*_2_ln *φ*_2_ + *χ n*_1_
*φ*_2_)

where *n*_1_ and *n*_2_ are the numbers of moles of the solvent and the polymer, respectively, *φ*_1_ and *φ*_2_ are the volume fractions of the solvent and the polymer, respectively, *χ* is the Flory–Huggins interaction parameter (determined by the energies of the intermolecular interactions), and *R* is the gas constant [10]. The parameter *χ* is considered dependent only on the temperature *T*. In this model, the *φ*_2_ at the critical point, *φ*_c_ → 0 when the polymer’s chain length approaches infinity [34].

However, PNIPAM does not obey the classical polymer behavior: its LCST only weakly depends on the polymer concentration (“flat” phase diagram) and molecular weight [34], and the transition is first-order [36]. There exist several approaches to describe the observed phase behavior of PNIPAM and its derivatives.

### 2.2. Type II Critical Phase Behavior of PNIPAM

Afroze et al. applied a different interaction parameter *g*(*φ*,*T*), depending on both the temperature and polymer concentration, instead of *χ* (*T*) [37]. In their model, the free Gibbs energy for a polydisperse polymer is expressed as

(3) $$\Delta G_{\mathrm{mix}}/\mathrm{NRT}=(\varphi_{1}/m_{1})\ln\varphi_{1}+\sum_{\left\{2i\right\}}{(\varphi }_{2i}{/m}_{2i})\ln\varphi_{2i}+g(T,\;\varphi_{2})\varphi_{1}\varphi_{2}$$

where *N* is the number of lattice sites, *m*_1_—number of sites occupied by solvent molecules, *m*_2_*_i_*_—_number of sites occupied by the *i*th species of the polymer, *φ*_2_*_i_* is the volume fraction of the *ith* species, *φ*_2_ = Σ *φ*_2_*_i_*; {*2i*} is the set of all the polymer species.

The *g* (*φ*_2_) dependence is assigned a polynomial form. Based on these assumptions, the chemical potentials for the solvent and the polymer are expressed as

(4) $$\Delta \mu_{1}/\mathrm{RT}=\ln\;(1-\varphi_{2})+[1-(1/{m_{n}}_{2})]\;\varphi_{2}+[g^{\left(0\right)}-(1-\varphi_{2})\;g^{\left(1\right)}]\;\varphi_{2}^{2}$$

(5) $$\Delta \mu_{2i}/\mathrm{RT}=\ln\;\varphi_{2i}+1-m_{2i}\varphi_{2}/m_{n2}-m_{2i}\varphi_{1}+[g^{\left(0\right)}+\varphi_{2}g^{\left(1\right)}]\;m_{2i}\;\varphi_{1}^{2}$$

where *g*^(i)^ denotes the *i*th order compositional derivative of *g*: *g*^(i)^ = $\partial^{i}g/\partial \varphi_{2}^{i}$.

Then, the spinodal and the critical conditions are read as:

(6) $$\varphi_{2}\;\{1/(1\;-\;\varphi_{2})\;-\;2\;g^{\left(0\right)}\;+\;2(1\;-\;2\;\varphi_{2})\;g^{\left(1\right)}\;+\;\varphi_{2}\;(1\;-\;\varphi_{2})\;g^{\left(2\right)}\}\;+\;m_{w2}^{-1}=0$$

(7) $$\varphi_{2}^{2}\;\{1/(1-{\varphi_{2})}^{2}-6\;g^{\left(1\right)}+3(1-2\varphi_{2})\;g^{\left(2\right)}+\varphi_{2}(1-\varphi_{2})\;g^{\left(3\right)}\}-m_{z2}/m_{w2}^{2}=0$$

where *m*_w2_ and *m*_z2_ are the mass and Z-average chain lengths of the polymer. Equation (6) determines the conditions for the spinodal, while the simultaneous satisfaction of Equations (6) and (7) determines the binodal.

The solutions of Equations (6) and (7) create the following three types of demixing behavior in a polymer–water system:

(1) Type I critical phase behavior with *φ*_c_ → 0 at infinite molar weight, corresponding to the classical Flory–Huggins Θ-behavior;
(2) Type II critical behavior with a single off-zero *φ*_c_ at non-Θ-conditions;
(3) Type III critical phase behavior with two off-zero *φ*_c_ and a *φ*_c_ → 0.

The experimental data showing that PNIPAM’s miscibility behavior demonstrates almost no dependency on the molar weight indicate that it belongs to Type II. The DSC data demonstrate that the positive enthalpy of demixing reaches a maximum at a certain concentration, consistent with the theoretical findings. Afroze et al. determine that, for infinite chain length, the critical conditions are *w*_2_*_c_* = 0.43 and *T_c_* = 299.5 K, a single off-zero critical point, without the classical *φ*_c_ → 0.

In a recent study by Dang and Nies [38] this theory is expanded to take into account the influence of end groups.

### 2.3. Cooperative Hydration

This theoretical approach developed by Okada and Tanaka [39] presents the concept of positive correlation between the adjacent hydrated groups in a PNIPAM chain. The positive correlation between the neighboring water molecules is sustained by hydrophobic isopropyl side groups.

In this model, the additional term of free energy due to correlated hydrogen bonding is introduced along with the common free energy of mixing from the Flory–Huggins theory. It is expressed through a modified *χ* parameter, *χ* + Δ*χ*, with Δ*χ* depending on the polymer concentration in a complex way.

The theory of cooperative hydration explains the observed phase diagrams and the phase transition in a narrow temperature region.

### 2.4. Two-State Theory of an Amphiphilic Polymer

Jarkova et al. [40] described the phase behavior of an amphiphilic polymer based on the two-state theory, considering hydrophobic (*H*) and hydrophilic (*P*) units. According to this model, the interaction part of the free energy is expressed as

(8) $$f_{\mathrm{int}}\;≅\;\frac{u_{\mathrm{HH}}}{2}\varphi_{H}^{2}+\frac{u_{\mathrm{PP}}}{2}\varphi_{P}^{2}+u_{\mathrm{HP}}\varphi_{H}\varphi_{P}+\frac{w}{6}\;{(\varphi_{H}+\varphi_{P)}}^{3}$$

where *u*_HH_, *u*_PP_ and *u*_HP_ are the second virial coefficients of interactions between the units, and *w* is the third virial coefficient that is the same for *H* and *P* units. While the hydrophilic units are soluble, *u*_PP_ > 0, the hydrophobic units are prone to attraction to each other, *u*_HH_ < 0.

The model predicts a first-order coil-to-globule transition in an amphiphilic polymer at a certain temperature, and may be applied to PNIPAM, as well, to explain its peculiar phase behavior.

### 2.5. Molecular Dynamics Simulations

There have been several molecular dynamics studies aimed at describing the phase behavior of PNIPAM. In [41], an isotactic 30-mer of PNIPAM was simulated below and above the LCST. The computations showed that the random-coil ensemble was more thermodynamically favored than the globule ensemble, by 21 ± 9 kJ/mol at T < LCST. At a temperature above the LCST, the globule ensemble’s minimum was 21 ± 8 kJ/mol lower than that of the coil ensemble. The energetic barrier of the coil-to-globule transition was calculated as 17 ± 10 kJ/mol.

Two MD simulation models were compared by Tavagnacco et al. in their study [42], TIP4P/2005 and TIP4P/Ice, the latter showing better agreement with the experimental findings. A recent study by Yagasaki and Matubayasi applied computations using the OPLS-AA model and its modified version in combination with the TIP4P/2005 water model [43]. Interestingly, the authors came to a conclusion that the coil-to-globule transition in PNIPAM is not a discontinuous first-order transition, but a continuous second-order transition consistent with the classical Flory–Huggins theory.

### 2.6. Mean Energetics of Water as the Factor Controlling the Phase Transition in PNIPAM

Based on their experimental studies of PNIPAM phase transition in the presence of kosmotropic additives (alcohols) and cononsolvency, Bischofberger et al. [44,45] considered the coil-to-globule transition in PNIPAM a problem purely of solvent thermodynamics.

The authors assume that the hydrophobic hydration of PNIPAM chains is controlled by the mean energetic state of the bulk water, while the interactions of hydrophilic groups with water play only a minor role. They conclude that the phase transition is only slightly dependent on the polymer chain behavior, and the hydrophobic hydration controlling the phase transition is mainly determined by the difference in the energy of the bulk and shell water.

### 2.7. Phase Transition in Crosslinked Gels

As mentioned above, the physical manifestation of the phase transition at the VPTT is entirely different from that for freely moving macromolecules. Since the chains are chemically anchored together in a gel, they cannot completely separate from each other or precipitate out of the liquid. Unlike the solubility change for an uncrosslinked polymer, for gels it is a volume change (swelling or collapse) manifested as the whole gel expansion or shrinkage. While for the solution each polymer coil becomes a globule, for the 3D network it means mesh shrinkage and water expulsion.

The conventional approach towards the thermodynamic description of crosslinked PNIPAM hydrogels is based on the Flory–Rehner model [46,47,48], which states that the total free energy change Δ*G*_total_ in a swelling 3D hydrogel consists of two additive contributions—the mixing component Δ*G*_mix_ (driving force for the dissolution) and the elasticity component Δ*G*_elast_ (restraining force for the network):

(9) Δ*G*_total_ = Δ*G*_mix_ + Δ*G*_elast_

When these two forces balance each other in a 3D network, the gel can no longer swell.

Afroze et al. [37] postulate that, in the case where the parameter *g*(*φ,T*) is not affected by crosslinking, the Gibbs free energy of mixing for a 3D network is constituted by two components:

(10) Δ*G*/*NRT* = (*φ*_1_*/m*_1_)ln*φ*_1_ + *g*(*T*, *φ*_2_) *φ*_1_
*φ*_2_ + Δ*G*_elast_

where Δ*G*_elast_ is the elastic contribution determined by the polymer volume fraction at network formation and the density of crosslinks.

Afroze et al. show that the phase behavior of PNIPAM hydrogels is the same as that of PNIPAM solutions. However, for a 3D hydrogel network, there is a three-phase equilibrium, involving a highly swollen phase, a collapsed phase and a pure solvent. In accordance with the Gibbs phase rule, it is invariant, leading to a concentration jump and a discontinuous swelling behavior at the VPTT. This interference of swelling with the miscibility gap originates from the Type II phase behavior of PNIPAM.

The mixing part of the Gibbs free energy is often considered from the viewpoint of the *χ* parameter dependency not only on the temperature, but also on the polymer concentration [46,47]. For example, in Paulin et al. it is presented as

(11) *χ* (*T, φ*) *= χ*_1_(*T*) + *χ*_2_(*φ*)

where *χ*_1_(*T*) = $\frac{{∆H}_{\mathrm{mix}}\;-T{∆S}_{\mathrm{mix}}\;}{k_{B}T}$, and *χ*_2_(*φ*) is approximated as a polynomial function of *φ*.

For the description of the elastic part, such models as the phantom network theory and affine network theory are applied [48].

The experimental studies by Grinberg et al. [49] showed the influence of the crosslink density on the thermodynamic functions of the VPTT in PNIPAM hydrogels: the transition becomes broader, and the VPTT decreases with increasing the crosslink density; at the same time, the enthalpy, entropy, and heat capacity increment of the transition remain unchanged.

Microgels are generally more favorable for use as injectable hydrogels than bulk hydrogels are. The swelling of microgels can also be modeled by the Flory–Rehner theory [50,51,52]. However, as highlighted by Lopez and Richtering [50], the PNIPAM-based microgels are distinct from the corresponding macrogels with a small surface charge, heterogeneous density of crosslinks, and a likely varying polymer volume fraction at network formation.

## 3. Gelation and Hydrogel Phase Behavior Controlled by the Coil-to-Globule Transition

### 3.1. Mechanisms of Coil-to-Globule Transition

It is conventionally believed that gelation in PNIPAM solutions occurs due to the coil-to-globule transition. The mechanism of the coil-to-globule transition has been extensively investigated both in theoretical [33,53] and experimental [54,55,56,57] studies.

Although the details of the mechanism description depend on the used approach, the general thinking is that the phase transition at the LCST proceeds via the cooperative rearrangement of the PNIPAM chains’ hydration shells. At T < LCST, PNIPAM macromolecules adopt an extended conformation and contain a large amount of bound water, as much as 11 water molecules per polymer unit [58].

**Figure 3:**
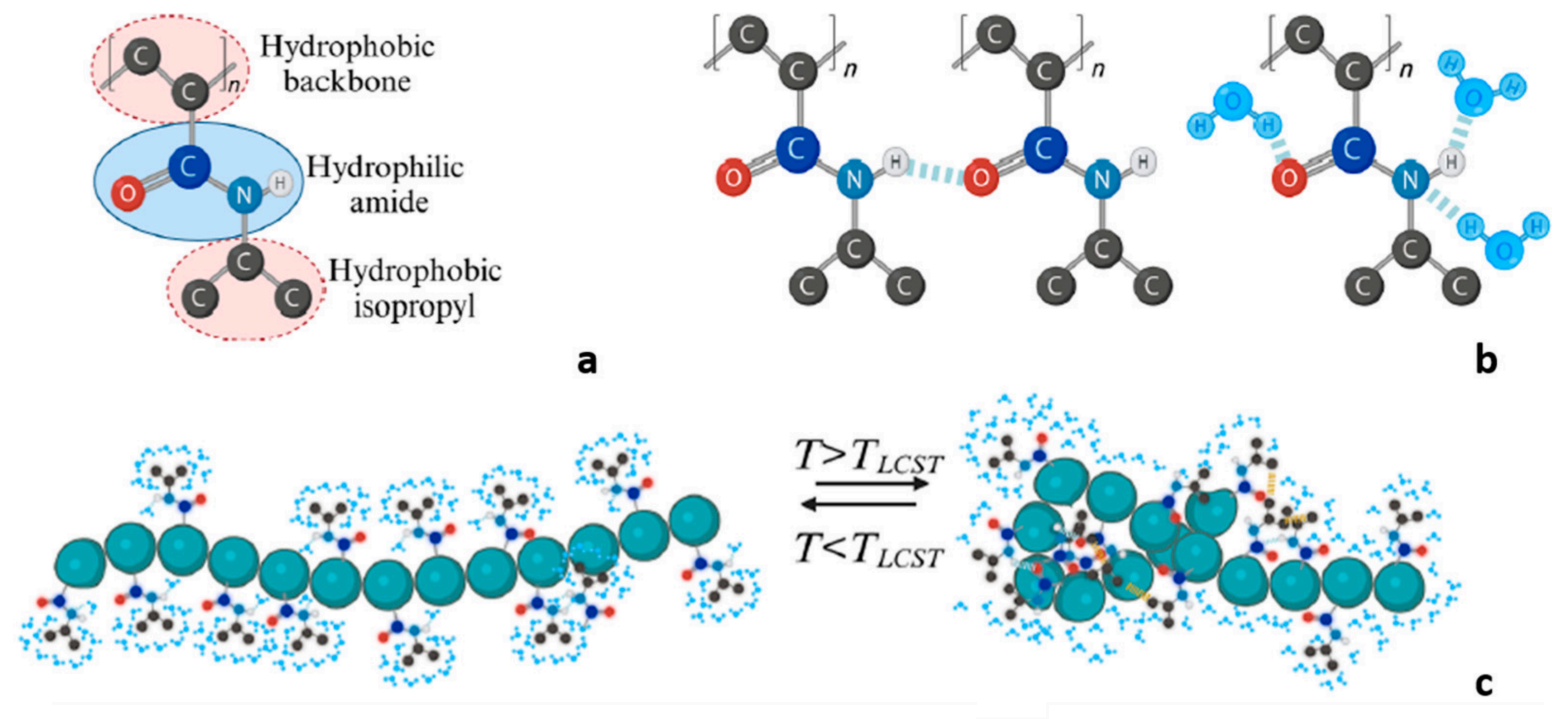
Coil-to-globule transition in a single PNIPAM chain. (**a**)—a repeat unit of PNIPAM with a hydrophobic backbone and isopropyl group and a hydrophilic amide group; (**b**)—hydrogen bonding between two PNIPAM macromolecules and between a PNIPAM macromolecule and water; (**c**)—an extended PNIPAM macromolecule below the LCST with “water cages” and the collapsed macromolecule above the LCST with “water cages” mostly destroyed [33]. Adapted from Macromolecules under Creative Commons Attribution 4.0 International License.

The chain hydration includes the formation of hydrogen bonds between the hydrophilic moieties in PNIPAM and molecules of water and “water cages” surrounding the hydrophobic groups, which form to separate the hydrophobic isopropyl groups from the bulk of water (Figure 3), giving rise to the hydrophobic hydration of PNIPAM [33]. Upon heating to the LCST, several steps take place, according to [33]: (1) thermal fluctuations intensify, destabilizing the hydrophilic interactions and hydrophobic hydration and causing water disordering; (2) the hydrophobic fragments of macromolecules, stripped of their hydration shells, begin to interact with each other; (3) hydrophobic clusters are formed to minimize exposure to the aqueous medium; (4) hydrogen bonds between the polymer’s hydrophilic groups and water break, and new hydrogen bonds are created between adjacent hydrophilic groups in PNIPAM. The latter may play the role of physical crosslinks in the hydrogel. The cooperative dehydration of the polymer chains results in their collapse with expulsion of bound water. However, even above the LCST PNIPAM is still far from total dehydration [59].

The experimental studies confirm the conclusions of molecular dynamics computations. Futscher et al. performed an FTIR spectroscopy study of PNIPAM in water at different temperatures and demonstrated that the hydration-dehydration process at the transition temperature involves both the hydrophobic (methyl and methylene) groups and amide groups participating in the formation of hydrogen bonds [55]. Wu et al. used direct time-resolved spectroscopic studies of a PNIPAM hydrogel near the VPTT to reconstruct the reaction coordinate of the chain collapse [56] and showed that the dehydration process was initiated by the hydrophobic groups at a time of 107 ± 37 ns with the amide group dehydration as the second step at 360 ± 85 ns. A Raman multivariate curve resolution spectroscopy study performed by Mochizuki and Ben-Amotz [57] revealed that, upon heating to the LCST, the hydration shell lost its tetrahedral order and strong hydrogen bonding to adopt a less ordered water packing. The time-resolved experiments detected also that this rearrangement took place after the clouding point was reached and the initial polymer aggregates ripened.

### 3.2. Mechanisms of Physical Gelation in PNIPAM Solutions

The clustering between macromolecules above the transition temperature is responsible for the formation of a physical gel from a PNIPAM-based solution (sol). Knowledge about the precise ways of its formation is scarce.

**Figure 4:**
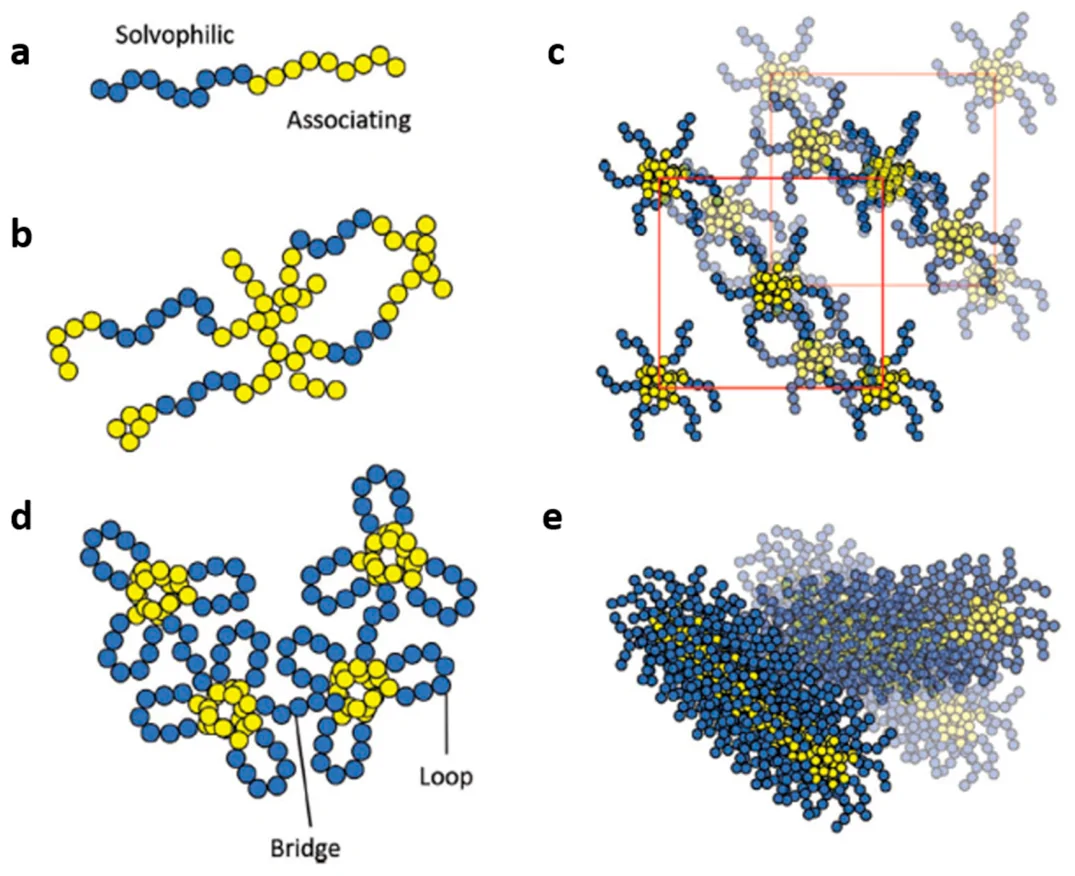
Mechanisms of physical gelation in block-copolymers containing hydrophilic blocks and thermoresponsive blocks [10]. (**a**)—a single chain with hydrophilic and thermoresponsive (associating) blocks; (**b**)—gelation via physical entanglement; (**c**)—gelation via the association of micelles with the formation of liquid crystalline structures; (**d**)—ABA triblock copolymers may form flower-like structures bridged to a percolating network; (**e**)—elastic network formed by cylindrical micelles. Reproduced from Advanced Functional Materials under Creative Commons Attribution 4.0 International License.

The most well-known mechanism is gel formation from micelles, suggested for block-copolymers of PNIPAM with more hydrophilic comonomers [1,10]. Similar to the critical conditions for micellization, the critical gelation concentration and critical gelation temperature are the necessary conditions for micelles to start forming a gel [1]. Cook et al. list a number of gelation mechanisms for diblock- and triblock-copolymers (Figure 4). First, their gelation may proceed via simple chain entanglement without micelle formation. Second, macromolecules may assemble themselves into micelles which may further pack together, creating liquid crystalline ordered structures. Third, ABA triblock copolymers may form flower-like structures bridged to a percolating network. Fourth, micelles may also arrange themselves in rows creating cylindrical structures held together by interactions between cylinders or physical entanglement to produce elastic networks.

Suvarna et al. suggest three different mechanisms for micelles to pack themselves into a gel: (a) individual micellar packing, inherent in low-molecular-weight diblock and ABA triblock copolymers, (b) inter-micellar bridging packing occurring in BAB-type (hydrophilic-hydrophobic-hydrophilic), BAC triblock, and multiblock copolymers, and (c) micellar corona collapse packing [1]. The latter mechanism is especially important for PNIPAM, since micelles of PNIPAM derivatives have a hydrophilic corona which undergoes a collapse above the LCST, leading to reduced repulsion between micelles, their aggregation, and thus to rapid gelation.

### 3.3. Mechanisms of Phase Transition in Crosslinked Hydrogels

Gels are different from both solid and liquid phases, their unique swelling/deswelling properties determined by the balance between the repulsive and attractive forces [6]. Thermoresponsive hydrogels exhibit dramatic changes in their volume, as well as in their wettability [60]. The data from Raman spectroscopy studies and molecular dynamics simulations showed that the isopropyl dehydration is responsible for the jumps in wettability [60].

Sun et al. applied 2D correlation infrared spectroscopy and a perturbation correlation moving window technique to study the precise thermodynamic mechanism of the chain collapse and revival in a poly(N-isopropylacrylamide) (PNIPAM) hydrogel [61].

The isosbestic points in ν_as_(CH_3_) and ν(C=O) overlaid spectra were investigated during heating and cooling of a PNIPAM hydrogel. The isosbestic point was absent when heating the hydrogel, which implies that the chain collapse proceeds either through multiple intermediate states or as a continuous process. During the cooling part of the thermal cycle, both bands exhibited isosbestic points; thus, the PNIPAM chain revival proceeded in one step with two single states (hydrated and dehydrated). In contrast, a 20% solution of PNIPAM showed isosbestic points both upon heating and cooling.

It was shown that, upon heating the hydrogel to the VPTT, the first step was the backbone response with the chain collapse followed by the expulsion of water molecules beyond the hydrogel borders. The formation of hydrogen bonds between amide groups instead of amide–water hydrogen bonds acted as a driving force for the chain collapse. Isopropyl pendants reacted later than the backbone did. During cooling, water molecules first moved into the network, being the driving force for the following backbone response and chain revival. Isopropyl groups, again, reacted after the backbone.

Based on the NMR study results, Burba et al. describe dehydration of PNIPAM hydrogels at the VPTT as a two-stage process [62]. At the first stage, the polymer backbone reacts by the rearrangement based on hydrophobic interactions. At the second stage, hydrogen bonds of hydrophilic fragments are disrupted, followed by the destruction of “water cages” around the hydrophobic moieties. Finally, stable hydrophobic bonds are formed between the exposed isopropyl groups, leading to the fast process of dehydration and association of hydrophobic fragments. The polymer rehydration starts from the rupture of hydrophobic bonds; then, the hydration shells are formed around the polymer chains.

**Figure 5:**
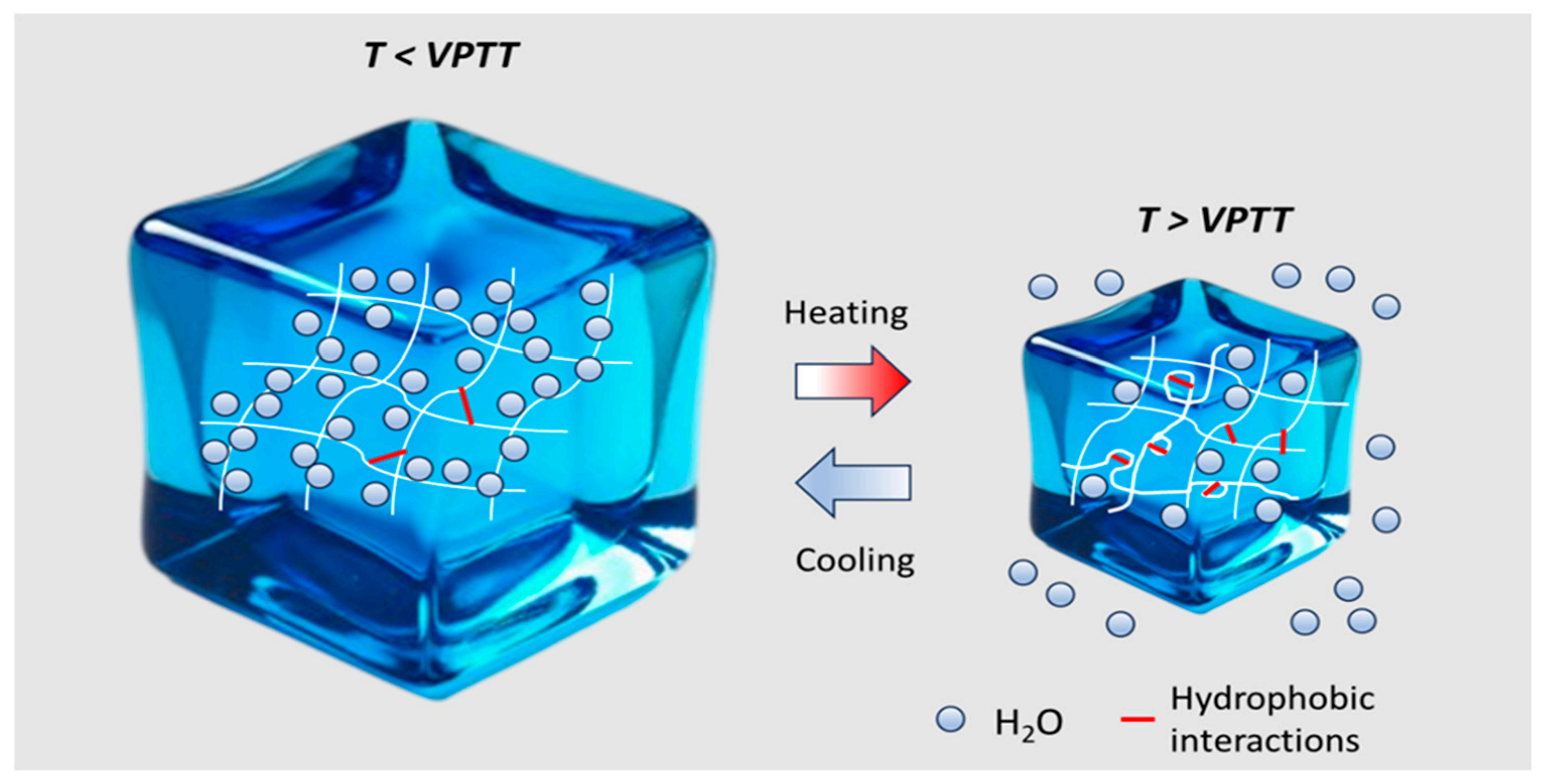
Mechanism of the coil-to-globule phase transition in a crosslinked thermoresponsive hydrogel. At T < LCST, the polymer chains in the 3D network adopt an extended conformation forming strong hydrogen bonds with surrounding water molecules. Above the coil-to-globule transition temperature, the network collapses, polymer chains are mostly stripped from their hydration shells, and hydrophobic interactions dominate. The water molecules from the hydration shells of polymer chains are released beyond the shrunk hydrogel volume.

Figure 5 summarizes the processes taking place during the phase transition in PNIPAM-based thermoresponsive hydrogels.

## 4. Gelation Controlled by Coil-to-Globule Transition: Some Examples

In spite of the dominating concept of PNIPAM gelation due to the coil-to-globule transition, in fact, there have not been many publications in which it is tested experimentally. In the majority of studies, including those mentioned in the numerous reviews, it is simply implied that the PNIPAM-based polymer under study undergoes gelation via this mechanism. To find the cloud point temperature, turbidity measurements or DSC studies are routinely performed, while rheological studies are commonly used to monitor the temperature course of gelation. It should be noted that, most frequently, only one technique is used by researchers, and both processes (gelation and coil-to-globule transition) are assigned to the measured temperature. Another approach, commonly seen in the studies dedicated to the medical applications of PNIPAM-based polymers, involves measuring the system’s properties well below and well above the transition temperature to demonstrate its liquid and gel state, respectively, only at those two characteristic temperature points.

However, one can find some publications on PNIPAM derivatives, which clearly demonstrate the coil-to-globule transition and the sol–gel transition being the same process for a system under study, using two techniques to monitor both transitions.

Rijns et al. designed a benzene-1,3,5-tricarboxamide (BTA-EG4) supramolecular system for potential use in tissue engineering by incorporating a poly(N-isopropylacrylamide)-functionalized (BTA-PNIPAM) moiety [63]. The cloud point temperature was measured by the turbidity studies at 600 nm and found to be 24 °C, 8 °C lower than the LCST of neat PNIPAM. The rheology studies found the same temperature at which the system became a hydrogel.

Ma et al. synthesized a methacrylate-polylactide copolymer and copolymerized it with NIPAM and 2-hydroxyethyl methacrylate (poly(NIPAM-co-HEMA-co-MAPLA)) in order to prepare bioabsorbable thermoresponsive hydrogels [64]. Optical absorption curves and DSC studies were used to measure the cloud points of the copolymers. The cloud points coincided with the temperatures at which both G′ and G′′ abruptly changed. Although G′′ remained higher than G′ even above the transition point, the authors still believed that the sol–gel transition took place, based on the totality of the samples’ properties.

In the study [65], two thermoresponsive graft copolymers of PNIPAM with poly(N,N-dimethylacrylamide) with inverse topologies were prepared: (PNIPAM-g-PDMA) consisting of a PNIPAM backbone with PDMA pendants and PDMA-g-PNIPAM with a PDMA backbone and PNIPAM pendants. Both DSC and rheology studies were performed with solutions of the copolymers. It was shown that the PDMA-g-PNIPAM copolymer had the temperature of gelation consistently lower than the LCST by 4–5 °C. At the same time, the copolymer with inverse topology, PNIPAM-g-PDMA, had the temperatures of gelation slightly lower than, equal to or higher than the LCST, depending on the concentration.

Similar temperatures of gelation and coil-to-globule transitions were measured for PNIPAM-grafted nanocellulose hydrogels in [66], using the rheology and turbidity studies, respectively.

Dong et al. prepared a novel hydrogel based on PNIPAM and poly(γ-glutamic acid) loaded with superoxide dismutase for trauma treatment [67]. The phase transition temperature of 28.2 °C was obtained both in the DSC and rheology studies.

Teodorescu et al. studied thermogelation of an injectable hydrogel with a semi-interpenetrating network structure, prepared by combination of PNIPAM with sodium alginate [68]. The temperature at which viscosity and G′ sharply increased, considered as the gelation temperature, corresponded to the temperature of the maximum endothermic effect determined by DSC.

A series of semi-IPN hydrogels based on PNIPAM copolymerized with itaconamic acid were synthesized and investigated in the study [69]. The LCST of the hydrogels was in the narrow range of 32.0–32.7 °C, while temperatures in the rheological studies were slightly higher than VPTT.

For triblock PNIPAM-b-PVP-b-PNIPAM copolymers [70] the points at which G′ exceeded G″ were those of the cloud points.

Nguyen et al. [71] performed a study on the understanding the influence of the polymer chain architecture on the properties of a forming hydrogel. They studied three types of architectures—a linear PNIPAM and two core–shell structures with PNIPAM shells. The cloud point temperatures were measured by DSC, and gelation was monitored via rheology. Neat PNIPAM formed hydrogels only at the highest concentration of 20%, while the two PNIPAM-grafted constructs formed gels at a lower concentration (15%). The transition temperature measured by DSC coincided with the gelation temperatures determined by the rheology studies.

The influence of hydrophilic silica nanoparticles on the microstructure, rheology, and stability of PNIPAM solutions was studied in [72]. PNIPAM with two molecular weights and three different loadings of nanoparticles was investigated both by DSC and rheology. The rheological transition temperatures were found close to those determined by DSC.

The mentioned studies present evidence that the coil-to-globule transition is the process responsible for gelation in the corresponding PNIPAM-based compositions. Such combined measurements appear highly desirable when considering injectable hydrogels, since, as will be shown in the following sections, this is not always the case, and different mechanisms may lead to the formation of a hydrogel.

The above considerations of the phase diagrams and the mechanisms of gel formation imply that the injectable hydrogel design must take into account the following factors. First, the chemical structure of a PNIPAM-based polymer is closely associated with the temperature of the coil-to-globule transition and the process of gelation. The presence of hydrophobic units in the PNIPAM structure reduces the transition temperature, making gelation inside the body favorable, while hydrophilic units increase the LCST that may hinder gel formation inside the body. The polymer’s molecular architecture also determines whether it can form micelles, since such structures are believed to form physical gels more easily. Then, a higher molecular weight (chain length) facilitates gel formation due to greater intermolecular association and entanglement, but at the same time increases the initial solution viscosity leading to poorer injectability. The balance of these two conditions should be sustained. In 3D crosslinked networks, the crosslinking density influences the VPTT, width of the transition and the mechanical properties of the resultant hydrogel that should be controlled depending on the hydrogel application. The concentration of the polymer should exceed the critical gelation concentration for the system to be capable of gelation.

## 5. Effects of Salts on the Phase Transition of PNIPAM-Based Polymers

The effect of ions on the coil-to-globule transition and gelation in PNIPAM-based hydrogels appears crucial for a number of reasons. First, the most important biomedical applications of injectable hydrogels involve operation in biological buffers, growth media and biological fluids, which can cause dramatic changes in the transition temperatures [10]. Second, the introduction of ions is an efficient way to modulate these processes, as well as the properties of formed hydrogels, without addition of crosslinkers, various modifiers, and special equipment for processing [2,10,73,74].

The basic influence of ions on the coil-to-globule transition in PNIPAM-based polymers, or the Hofmeister effect, is associated with their competition with PNIPAM chains for molecules of water [2,10]. Ions are arbitrarily divided into “kosmotropes” or “chaotropes” by their interactions with the hydration shells of polymers. This division stems from the trend known as the Hofmeister series, in which salts are arranged by their ability to precipitate proteins from aqueous solutions [75]. The general tendency remains similar for thermoresponsive polymers, including PNIPAM. Kosmotropes can reduce the phase transition temperature through the disruption of hydrogen bonds between water and polymer chains, thus promoting the hydrophobic interactions and leading to polymer precipitation (“salting-out” effect). On the contrary, chaotropes stabilize the hydrogen bonds of polymers resulting in the “salting-in” effect and elevated transition temperatures. Kosmotropic salts strongly interact with water and are surrounded by dense hydration shells, while chaotropes interact weakly with water and form weakly bound hydration shells [76].

The typical order of anions and cations in the Hofmeister series is the following [75,77]:

CO_3_^2−^ > SO_4_^2−^ > S_2_O_3_^2−^ > H_2_PO_4_^−^ > F^-^ > Cl^−^ > Br^−^ ≈ NO_3_^−^ > I^−^ > ClO_4_^−^ > SCN^−^

Zn^2+^ > Mg^2+^ > Ca^2+^ > Li^+^ > Na^+^ > K^+^ > Cs^+^ > NH_4_^+^

The left side of the series represents kosmotropes; the ions on the right are chaotropes. These properties are more pronounced for anions than for cations [62,75].

The commonly observed tendency for PNIPAM represents a linear decrease in the cloud point with increasing kosmotropic salt concentration [75,76,78]. For a chaotropic anion, the dependency is non-linear, e.g., for I^−^ and SCN^−^ the cloud point first increased with concentration, exhibiting a maximum [76]. The strong interaction between a kosmotropic ion and water leads to the formation of a thick hydration shell around the ion, stripping the polymer chain from some of its hydration water and promoting interactions between the hydrophobic fragments of the polymer, thus leading to the salting-out effect. In contrast, chaotropic ions demonstrate a salting-in effect; however, the second salting-out effect may be observed at a higher concentration of a chaotropic salt due to the excess hydration and saturation of amide groups [76].

**Figure 6:**
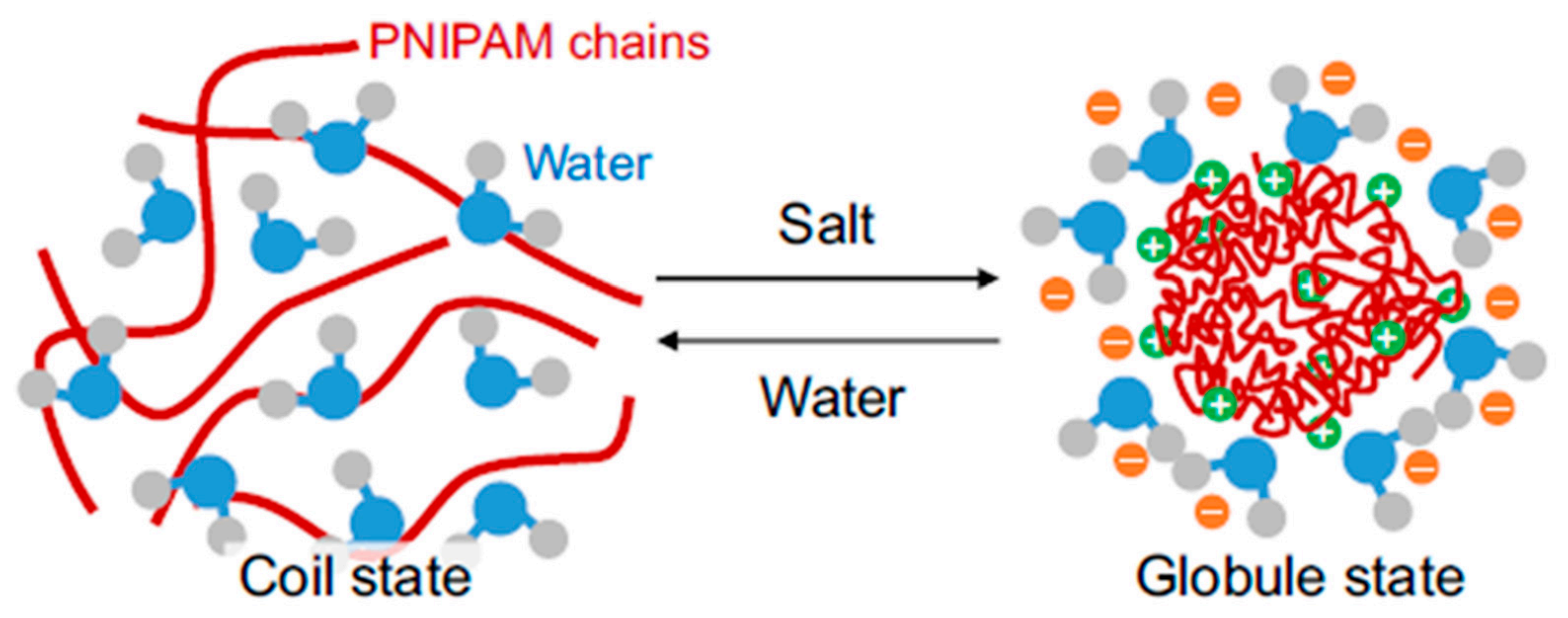
Salting-out of PNIPAM molecules in the presence of kosmotropic anions [73]. Reproduced from Nature Communications under a Creative Commons Attribution 4.0 International License.

Density functional theory computations applied to a PNIPAM chain unit showed that the hydrogen atom of the amide group was almost exclusively responsible for the formation of hydrogen bonds, while the C=O group and nitrogen atom did not have significant differences in the electron densities [76]. Three basic mechanisms associated with the influence of salts on PNIPAM’s phase behavior have been suggested [62,75,77]. The first mechanism involves hydrogen bonds formed by amide groups of PNIPAM, which are hindered by the presence of an ion accumulating water molecules in its hydration shell. Through the second mechanism, a hydrated anion causes an increase in the surface tension of the cavity around the hydrophobic groups of the polymer, disrupting the formation of “water cages” and thus contributing to the polymer precipitation. These two mechanisms primarily control the salting-out action of kosmotropic ions on a PNIPAM chain (Figure 6). The third mechanism is related to the direct binding of an ion to the amide group, which enhances its polarization and affinity to water. The third mechanism is mostly responsible for the salting-in effect of chaotropic salts.

These three mechanisms were analyzed based on the experimental findings in [75]. Light scattering experiments showed that kosmotropic salts demonstrated linear dependencies with a complex behavior. At low salt concentration, a single linear dependence was observed, but, starting from a certain concentration, the transition became a two-stage process, with two different slopes. The concentration dependence for chaotropic salts was non-linear.

The LCST dependencies on the salt concentrations were modeled based on three terms—a constant (LCST without salt), a linear concentration term, and a Langmuir isotherm characterizing direct binding of anions to the polymer. It was shown that the slope for the lower-temperature transition in the presence of kosmotropic anions was correlated with the hydration entropy of anions. Thus, this phase transition was assigned to the amide group dehydration due to weakening of its interaction with water resulting from water polarization by the highly hydrated anions. The high-temperature transition was attributed to the weakening of the hydrophobic hydration, since the LCST change was found to be correlated with the anions’ surface tension increment. Subtraction of the constant and the linear term from the cloud point–concentration plots produced Langmuir-shaped binding isotherms for chaotropic salts. The strength of binding series was obtained as ClO_4_^−^ > SCN^−^ > Br^−^ > NO_3_^−^ > Cl^−^. The highly hydrated kosmotropic species showed the weakest binding to amide groups. Thus, it was shown that amide dehydration was the primary mechanism for kosmotropes, while ion binding to amide groups mainly controlled the effect of chaotropes.

The salt effect on the thermodynamics of PNIPAM phase transition has also been investigated in several studies.

Using NMR spectroscopy, Burba et al. studied the thermodynamics of the phase transition in crosslinked PNIPAM hydrogels in the presence of NaCl and CaCl_2_ [62]. Two stages of the phase transition were derived from the van’t Hoff plots, both processes being endothermic. Based on the enthalpy values, it was suggested that stage I was determined by the formation of hydrophobic bonds, and dissociation of hydrogen bonds between the polymer and water was assigned to stage II. The entropy changes for stage II were greater due to the massive release of water molecules from the hydration shell of the polymer.

The experiments showed that the enthalpies for stage I of the dehydration process dropped from 3.4 kJ/mol in water to 1.5 kJ/mol in 150 mM NaCl and 1.8 kJ/mol in 150 mM CaCl_2_, consistent with the salt effects on hydrophobic interactions. The entropy changes for stage I were also significant −4.9 and 5.9 J/mol ·K in NaCl and CaCl_2_ solutions, respectively, vs. 11.2 kJ/mol·K in water. In contrast, only slight changes in the enthalpy and entropy due to the presence of salts were found for stage II. Thus, the salts had a greater impact on the formation of hydrophobic bonds upon the volume phase transition in hydrogels (stage I), while their influence on the thermodynamics of dissociation of amide–water hydrogen bonds was found almost insignificant. In the later study by the same group [78], more cations and their different concentrations were used to confirm and expand these findings.

Pan et al. built a thermodynamic model for PNIPAM hydrogels in salt solutions based on the model for PNIPAM in water developed earlier by Cai and Suo [47], adding the ionic concentration variable to the temperature and volume in the expression for the free energy [79]. Thus, the free energy was considered a function of temperature, volume and ionic concentration. Using the constructed model, the authors could predict the phase behavior of PNIPAM in salt solutions, in particular, the decrease in the phase transition temperature in the presence of kosmotropic salts depending on the salt concentration.

Pica and Graziano describe the effect of salts on the phase transition in PNIPAM based on the consideration of the solvent-excluded volume [80]. The difference between the energies to form a cavity for a coil and for a globule is believed to be the main parameter determining the magnitude of the effect. This difference is always positive since the water-accessible surface area is much larger for a coil than for a globule, and the solvent-excluded volume is also larger for a coil. Kosmotropic ions have a high charge density and are highly hydrated; they increase the density of the solution and boost the solvent-excluded volume effect. Chaotropes are characterized by a low charge density and are weakly hydrated. They directly interact with the polymer chain and prevent the formation of a globule.

Three thermodynamic contributions are considered to control the phase transition in the presence of salts: (1) upon the coil-to-globule transition, the solvent-excluded volume is reduced, resulting in the translational entropy gain for water molecules and for ions; (2) upon the coil-to-globule transition, the polymer chain is collapsed, so the conformational entropy of the polymer is decreased; (3) the loss in the enthalpy due to the rearrangement of interactions upon the chain collapse. When a salt is added, the density of the solution is changed, which impacts the difference between the energies to form a cavity for a coil and for a globule (solvent-excluded volume effect). For example, for a kosmotropic salt the increase in the solution density causes a positive solvent-excluded volume effect that stabilizes the globule state of the polymer. For a chaotropic salt, the direct binding to amide groups plays a major role, leading to stabilization of the coil state of the polymer and a slight increase in the transition temperature.

The effect of salts on the phase behavior of PNIPAM shows that, in the injectable hydrogels’ design, gelation and the hydrogel’s properties should be studied not only in pure water, but also in the medium to be used in the real conditions. In a general sense, other molecules and particles present in the medium should also be considered. The overall ionic strength of a physiologically relevant solution should lead to the LCST decrease, in accordance with the effects of the corresponding ions. The presence of proteins in the medium may differently affect the LCST depending on the polymer and the protein charges, as shown, e.g., in [81]. In addition, the protein corona forming around particles may hinder aggregation and thus affect the gelation. While PNIPAM itself is a neutral polymer with no potential pH influence, the presence of charged units may result in the pH influence on the coil-to-globule transition. The effect of macromolecular crowding on PNIPAM’s LCST in the absence and presence of kosmo- and chaotropic salts was studied in [82]. The presence of a crowding agent (PEG) itself caused a drastic decrease in the LCST of PNIPAM. Kosmotropic salts decreased the transition temperature independently of the presence of PEG, while the effect of chaotropic salts was different in the presence of the crowding agent. Live cells may affect the gel formation due to the presence of all the above factors added by the cell metabolism products and adhesion of the polymer at the cell surface.

Addition of salts appears an attractive way to govern the properties of PNIPAM hydrogels. Stiffer and more robust hydrogels may be obtained upon addition of kosmotropic ions, while addition of chaotropic ions gives rise to softer and more sensitive hydrogels [74]. For chemically crosslinked hydrogels, it was shown that the Young’s modulus did not depend on the salt type and concentration below the VPTT, while in the deswollen state, the tensile modulus decreased in the presence of salts proportionally to the salt concentration; thus, only the mixing part of the free energy, and not the elastic part, was affected by the presence of ions [83].

Various modalities based on controlling the salt type and concentration have been created in the fields of micro- and nanorobotics, polymersomes, nanomotors, ion sensors, and nanomedicine [74]. The use of the salting-out effect in 3D printing of thermoresponsive polymers allows reaching fast physical gelation at temperatures much lower than the LCST, at the same time preserving the solution injectability, without the use of chemical modifiers or additional processing [73].

## 6. Phase Behavior of PNIPAM Under Pressure and Mechanical Stress

Injection exerts mechanical stress upon a viscous PNIPAM solution, and after the polymer is injected, it undergoes the phase transition in the confined space; thus, the influence of pressure and mechanical stress is worth consideration. In addition, pressure variation may be used for the control of the hydrogel phase behavior [84,85].

The major effects of pressure on a PNIPAM solution are related to the changes in the state of water both in the bulk and in the hydration shell of the polymer [84]. Hydrogen bonds and hydrophobic interactions are mostly subjected to the action of high pressure. The bulk water becomes more ordered and closer to water in “water cages” at high pressure. The latter are more compressible than other water formations, such as hydration shells around hydrophilic groups. At elevated pressure, new denser water layers are formed around the hydrophobic groups leading to their higher hydration favoring the coil vs. globule state.

The pressure–temperature phase diagram of PNIPAM in an aqueous solution has a bell-like shape [84]: at lower pressures, the cloud point increases with pressure, then the maximum is observed at a certain pressure (about 60 MPa), and the following pressure elevation leads to the decrease in the transition temperature. Inside the “bell”, the system exists as one phase, while outside the bell it consists of two phases. This phase behavior has been described using the Hawley equation or the Clausius–Clapeyron equation [84,86].

As shown by DSC studies, the pressure increase causes the transition temperature to exhibit a maximum, the enthalpy of transition to decrease, and the transition to become wider. The transition-related partial volume increment of PNIPAM is positive at lower pressures and negative at high pressures, which is associated with the interplay between the hydrophobic hydration and hydrogen bond formation [84]. Small-angle neutron scattering, quasi-elastic neutron scattering, FTIR and Raman studies showed the following specifics of the PNIPAM phase transition under elevated pressure [84]:

- Hydrogen bonds between PNIPAM and water are weakened;
- The width of the transition increases—from ≈1 K at atmospheric pressure to more than 10 K at 130 MPa;
- The dehydration of the polymer chain during the transition under pressure is much less pronounced than that at atmospheric pressure;
- The mesoglobules formed above the transition temperature are small and compact in the low-pressure two-phase region, with Rg ~40–100 nm and a dense hydration shell; in the high-pressure region, mesoglobules form large aggregates with sizes >1 μm, containing a much higher amount of bound water.

The shear stress in a flow may also affect the phase behavior of polymer chains in solutions, as shown with the use of de novo nonequilibrium molecular dynamics [87]. A polymer globule swells and transforms into an extended coil at the LCST or higher temperatures, when the flow velocity exceeds a certain threshold value. For a 30-mer PNIPAM, the radius of gyration increases by approximately two times, while the end-to-end distance increases by 6 times, and the water-accessible surface area increases by 50%. At the same time, the water–polymer energy of interaction is lowered by approximately 8−10 kJ/mol. When the flow velocity is reduced below the threshold value, the extended coil transforms back to a globule.

Crosslinked hydrogels are affected by applied pressure in different ways [47]: in a hydrogel, attached to a stiff substrate, the phase transition temperature at the bottom and at the top may differ due to the constraint at one side; for a core–shell structure with the hydrogel inside and a stiffer polymer as a shell, the phase transition may even be suppressed; in contrast, in a fixed hydrogel rod, the phase transition may be induced by uniaxial stretching.

The pressure–temperature phase diagram for crosslinked hydrogels had the same appearance as that for solutions [86]. The coexistence curve had a bell-like shape; at low pressures, the VPTT increased with pressure (the initial part approximated by a linear dependence), then the saturation was achieved, and, starting from some pressure, the transition temperature decreased with increasing pressure up to very high pressures of >250 MPa. Inside the “bell”, the system was swollen, and outside the “bell”, it existed in the shrunk state.

Theoretical calculations based on the Flory–Rehner model showed that the phase transition temperature was not significantly influenced by the mechanical constraint of a hydrogel; however, the volume change at the phase transition was different for free, uniaxially constrained and biaxially constrained swelling [47]. The volume change decreased with the crosslink density of the hydrogel.

Under the constraint, the axial stress in a hydrogel rod rises upon heating in a temperature region near the VPTT, and the coexistence of two phase states (swollen and collapsed polymer domains present simultaneously for a sufficiently long time) is possible in the presence of mechanical loading [33,85]. The coexistence of two phases at the VPTT was observed experimentally [88], in the case when a fixed PNIPAM rod was heated above the VPTT, and in the case when a collapsed rod was uniaxially stretched at the VPTT. In both cases, stable coexistence of two states—collapsed and swollen—was registered. This phenomenon was explained in [33,85] by the local thermomechanics of a PNIPAM chain. Upon application of a significant local force, the intramolecular bonds in a collapsed chain are broken, the hydrophilic groups are exposed and open to hydration. On the scale of a network, the hydrated and extended chains cause the appearance of a swollen domain, inducing the local phase transition. In the case of a fixed swollen rod, heating above the VPTT causes local contraction of the chains due to the transition with the formation of collapsed regions. Over time, the equilibrium is reached in both cases with the whole rod being swollen or collapsed, respectively. The calculations showed that VPTT in a stretched rod increased as compared to the free rod.

In a core–shell structure, the main factor is the thickness of the stiff shell, which affects the discontinuity of the transition, but only slightly affects the transition temperature. However, the presence of a stiff shell can hinder the transition [47].

Takigawa et al. studied PNIPAM hydrogels under tension and found that their behavior differed under low and high tension conditions [89,90]. When the tension was low, the VPTT and degree of discontinuity increased with tension; however, when the tension was sufficiently raised, the tendency changed. The VPTT rose, but the transition became less pronounced. At a certain critical compression force, the first-order transition in PNIPAM transformed into a second-order transition. Starting from this critical force, the continuous volume change was observed vs. the discontinuity observed for a load-free gel or a gel under a lower load.

Though the pressure influence may appear minimal for applications related to injections, in the case of viscous fluids the injection pressure may be significant enough not to be neglected. The fundamental pressure studies can appear useful after the corresponding estimations based on the injection force, which is shown to depend on the fluid viscosity, inner diameter of the syringe needle, needle length and the inner diameter of the syringe barrel [91]. The shear stress arising during the injection may also impact the chain conformation; thus, the created hydrogel must be capable of rapidly recovering after the injection. The mechanical constraints upon the implantation should be considered, as well, as they may impact the hydrogel shrinking and lead to coexistence of two phases.

The combined pressure and salt effects may potentially be applied in the control of the phase transition/gelation of PNIPAM-based injectable compositions. Although there have been no such studies so far, as early as in 1999 Kunugi et al. demonstrated that the whole bell-like pressure–temperature phase diagrams of a PNIPAM isomer, poly(N-vinylisobutyramide), shifted either to slightly higher temperatures in the presence of chaotropic salts or to significantly lower temperatures in the presence of kosmotropic salts (they studied PNIPAM, as well, but presented no data on PNIPAM) [92]. Similar studies in the future may explore a combination of these two tools to tune the gelation, injectability and properties of PNIPAM-based hydrogels to benefit the injectable hydrogel design.

## 7. Gelation as a Separate Process from the Coil-to-Globule Transition

### 7.1. Three-Branched Phase Diagrams

Since PNIPAM-based polymers may form gels upon the coil-to-globule transition, it is the conventional thinking that gelation in such polymers occurs due to the coil-to-globule transition. However, many non-thermoresponsive polymers are capable of forming gels via other mechanisms, and generally there is no reason for PNIPAM-based polymers to not form gels via other mechanisms, as well. Constantinou et al. discuss a possibility of gelation being a separate process from the coil-to-globule transition and present another type of the phase diagram, a three-branched one [11]. Wang et al. warn against the default view of the physical gel formation in PNIPAM as the coil-to-globule transition, since there are thermoresponsive polymers in which these two transitions are separate [93].

A number of studies applied two techniques for the same temperature ramp and found that gelation in PNIPAM-based polymers under study appeared to be a different process with a separate branch on the phase diagram (these studies will be discussed in the next section). The following characteristic details have been found for such polymers. First, their gelation occurred at temperatures lower (at times significantly lower) than those of the coil-to-globule transition, with the formation of a transparent gel. The transparent gel became turbid upon the coil-to-globule transition, and, in some cases, the subsequent syneresis led to substantial water release. Second, the temperature of gelation significantly decreased with the polymer concentration, as opposed to the very weak dependency for the coil-to-globule transition temperature (“flat” phase diagram). Thus, with the polymer concentration increased, the region of the transparent gel existence below the coil-to-globule transition branch expanded. Third, no gel formation was observed below the critical gelation concentration, giving rise to the triple point on the phase diagram.

**Figure 7:**
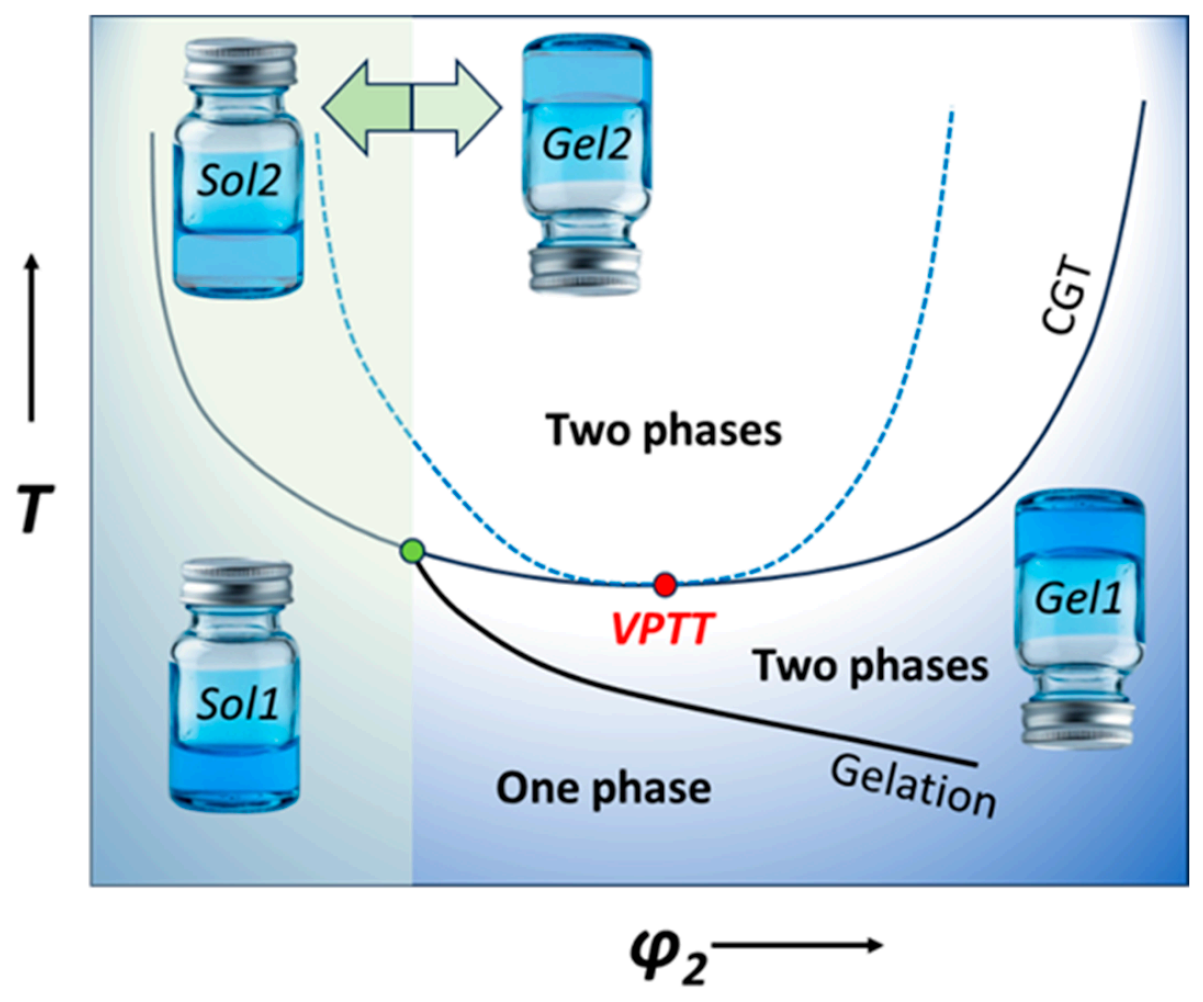
Phase diagram of a PNIPAM-based polymer with the process of gelation separate from coil-to-globule transition (*CGT*). *Sol1*—transparent sol, *Sol2*—turbid sol above the coil-to-globule transition temperature, *Gel1*—transparent gel below the VPTT, *Gel2*—turbid gel above the VPTT. The green band marks the region of concentrations too low for gel formation. The green dot marks the triple point of *Sol1–Gel1–Gel2* phases.

Thus, in general, the PNIPAM-based polymers with separate processes of gelation and coil-to-globule transition have a three-branched phase diagram which is schematically displayed in Figure 7. This phase diagram contains four regions—(1) transparent sol at temperatures below the gelation temperature (*Sol1*), (2) turbid sol at temperatures above the coil-to-globule transition temperature and below the critical gelation concentration (*Sol2*), (3) transparent gel above the gelation branch and below the coil-to-globule transition branch (*Gel1*), (4) turbid gel above the coil-to-globule transition branch (*Gel2*). The latter line corresponds to the volume phase transition between the two gels.

### 7.2. Experimental Examples of Gelation and Coil-to-Globule Transition Being Separate Processes

As of now, a number of observations of three-branched or even more complex phase diagrams have been reported for PNIPAM and its derivatives.

Nakano et al. studied isotactic-rich PNIPAM, measuring the cloud point temperatures from transmittance curves and sol–gel transition temperatures using the rate of a descending nickel ball [94]. They obtained a transparent PNIPAM gel at temperatures below the coil-to-globule transition and a turbid gel above the coil-to-globule transition temperature. The phase diagram plotted based on the two techniques’ results had three branches—the first branch for the transparent sol–turbid sol transition at low polymer concentrations, the second branch for the gelation (sol–gel transition), and the third branch for the transparent gel–turbid gel volume phase transition. Interestingly, the DLS-based power law exponent obtained for the physical gel of isotactic-rich PNIPAM was almost the same as one measured for chemically crosslinked PNIPAM. The authors believe that the structure of the physical gel network resembles that of the chemically crosslinked gel. No such behavior was registered for atactic PNIPAM.

Tanaka et al. experimentally observed four phases—transparent sol, turbid sol, transparent gel, and turbid gel—in stereoregular PNIPAM [95]. A thermodynamic model for stereoregular PNIPAM with varying fraction of meso dyads was built based on the competition of hydration and association processes. In the developed model, the isotactic segments formed crosslinks between macromolecules resulting in gelation. The calculated phase diagrams produced separate branches for the sol–gel transition and coil-to-globule transition spinodals, with their positions depending on the content of isotactic meso-dyads.

**Figure 8:**
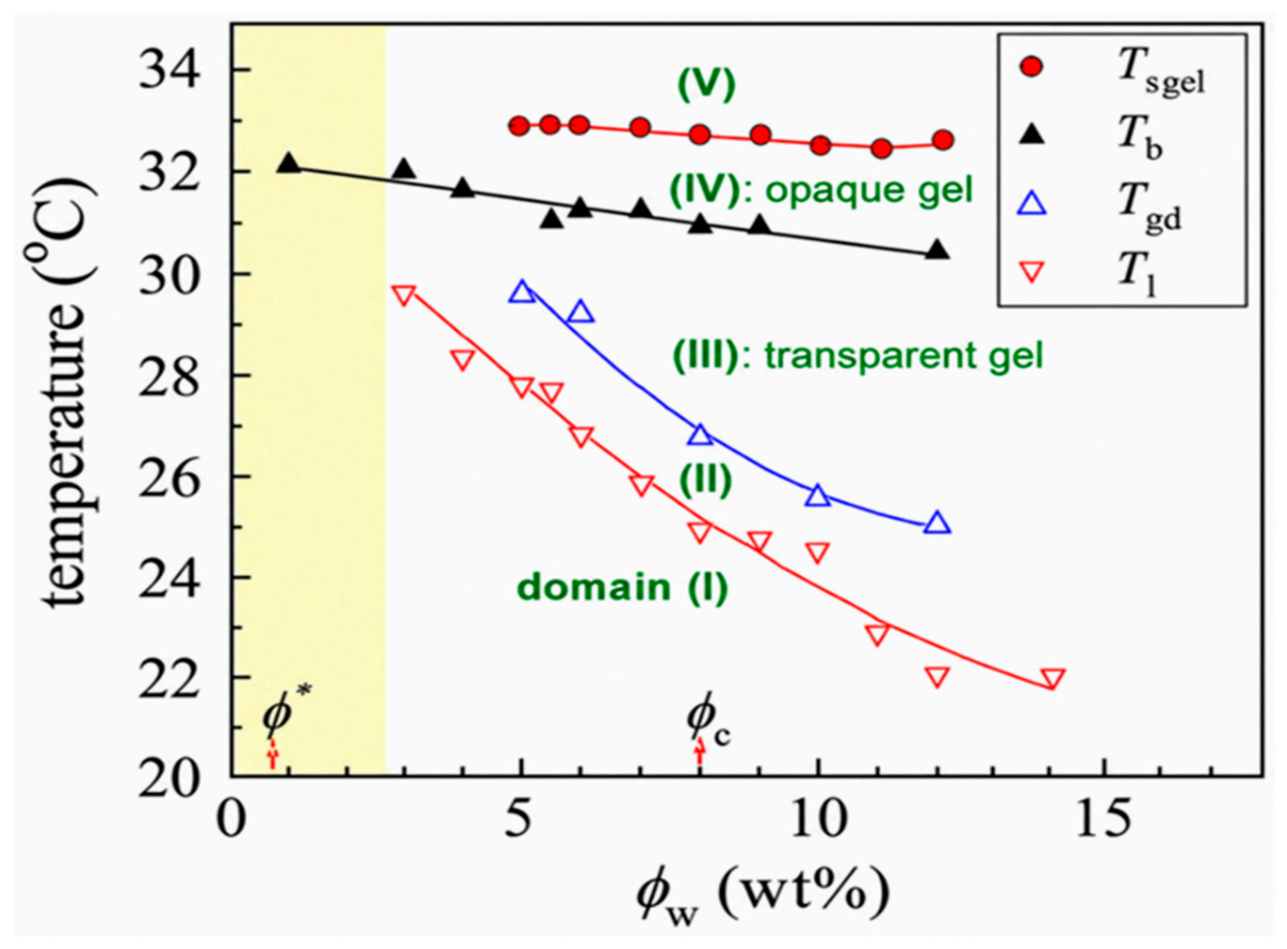
Phase diagram of atactic PNIPAM described in [97]. Five domains are identified in the quasi-equilibrium phase diagram depending upon the composition and temperature, namely, domains (I): T < T_1_, stable single-phase solution with the thermorheological simplicity, (II): T_1_ < T < T_gel_, the single-phase solution containing the interchain association subjected to profound concentration fluctuations, (III): T_gel_ < T < T_b_, transparent gel, (IV): T_b_ < T < T_s,gel_, opaque gel, and (V): T > T_s,gel_, unstable gel undergoing viscoelastic phase separation via spinodal decomposition. Reproduced from *Gels* under a Creative Commons Attribution 4.0 International License.

It is of note that chemically identical polymers may form transparent gels in one experiment and not undergo gelation in another experiment. Nakano et al. [94] and Tanaka et al. [95] believed that tacticity was a crucial factor for PNIPAM; however, later studies by Wang et al. demonstrated formation of transparent gels in atactic PNIPAM [93,96,97,98,99]. In these extensive studies, the gel point of atactic PNIPAM was measured through frequency-independent loss tangent (Winter–Chambon criterion) in the oscillatory shear measurements, while the cloud points were determined via the light transmittance studies. Three-branched phase diagrams were obtained for atactic PNIPAM, and additionally the authors defined a “pregel” state, in which physical “crosslinking sites” begin to appear (Figure 8). Based on the phase behavior and structural studies, the authors modeled the formation of a gel in such systems below the coil-to-globule transition temperature, and also modeled nucleation and growth and spinodal decomposition in transparent gels.

Schoolaert et al. researched electrospinning of low-molecular-weight (30 kDa) PNIPAM from aqueous solutions and found that the polymer viscosity at 25 °C substantially rose for semi-dilute solutions, while its cloud point was determined at 31 °C independently of the polymer concentration [100]. The polymer formed transparent gels at temperatures lower than the coil-to-globule transition temperature. The gel point was observed to essentially decrease with the concentration, reaching a ±17 °C difference at 12 wt.%, while a 14 wt% solution was already a transparent gel at a temperature as low as 10 °C. The transparent gel transformed into an opaque gel upon heating to the temperature of the coil-to-globule transition.

The presence of transparent gels at temperatures lower than the coil-to-globule phase transition temperatures was also observed in PNIPAM copolymers of various architectures. Although block architecture is well-known to promote gelation, random and graft copolymers were also shown to form transparent gels at temperatures below the LCST.

In the study by Motokawa et al. the phase behavior of diblock copolymers of PNIPAM with PEG was investigated [101]. The cloud points were measured by transmittance studies, and gelation was monitored visually with a vial inversion method. Complex phase diagrams were obtained, in which the presence of transparent gels depended on the PNIPAM–PEG ratio. Another diblock copolymer of PNIPAM, containing a glucose-bearing comonomer, demonstrated gelation at temperatures much lower than the cloud points in the study by Tang et al., performed with the use of the tube-inverting method [102].

A complex phase behavior of random PNIPAM-butyl acrylate copolymers with different molecular weights, synthesized by RAFT copolymerization, was observed by Li and Thompson [103]. At low concentrations, the polymer solutions demonstrated only a coil-to-globule transition with the appearance of an opaque sol. In the semi-dilute regime, formation of transparent gels was registered. The region of the transparent gel phase expanded with the copolymer concentration, molecular weight and fraction of butyl acrylate units. Interestingly, a block copolymer of PNIPAM with butyl acrylate did not form transparent gels. The transparent gels demonstrated excellent elasticity, stability and self-healing properties. One more phase was identified by the authors—a dehydrated gel phase (precipitate), which formed upon further heating of the opaque gel.

Wintgens and Amiel showed that, upon heating, a PNIPAM copolymer containing adamantyl side groups gelled prior to the coil-to-globule transition, whereas linear dodecyl-containing PNIPAM underwent gelation and a coil-to-globule transition at the same temperature [104].

In our recent study, we described three-branched phase diagrams for well-defined graft copolymers of PNIPAM with polylactide, synthesized by RAFT copolymerization [105]. The coil-to-globule transition was studied by DSC, and gelation was monitored by rheological studies. We found that the gel formation and coil-to-globule transition were separate processes in semi-dilute polymer solutions of the copolymers, and the formation of a transparent gel preceded the coil-to-globule transition upon heating. We observed all the characteristic features of this type of thermoresponsive polymers mentioned above, including the strong concentration dependence of gelation vs. weak concentration dependence of the coil-to-globule transition, volume phase transition from a transparent to opaque gel, and the presence of the critical gelation concentration.

Abou-Shamat et al. recently reported a system composed of a PNIPAM-PEG-PNIPAM copolymer added to poloxamer 407 in tertiary mixtures with water [106]. They showed that such mixtures of two thermoresponsive polymers could form transparent gels at temperatures lower than those of the coil-to-globule transition, with the subsequent gel–gel transition upon further heating to the VPTT, while individual polymers had only one phase transition.

The existence of a three-branched phase diagram cannot currently be predicted for a certain injectable system. Thus, it appears reasonable that more than one experimental technique should be applied to establish the gelation temperatures and coil-to-globule transition temperatures for PNIPAM and its derivatives when creating an injectable hydrogel. UV-Vis turbidity and DSC studies are commonly used as gold standards for tracking the coil-to-globule transition, though sometimes the appearing opacity is observed visually. For gelation, rheology studies are used routinely; the tube inversion method is applied more rarely, and the technique based on the rate of a metal ball sinking is described in [94]. When using rheology studies, one should be cautious about using a single G’=G” point on a temperature sweep curve as a gelation temperature, since this approach has its limitations, such as the gel point dependence on the frequency [107]. Application of a more rigorous method such as applying the Winter-Chambon criterion (independence of the loss tangent from the frequency at the true gel point) is validated in the latter case.

### 7.3. Mechanism of Gelation at Temperatures Below the LCST

Formation of a 3D network in a polymer solution presumes the presence of sufficiently strong inter- and intramolecular interactions. In addition, a certain threshold concentration, at which polymer chains start to touch each other, must be reached. This threshold defined as a critical overlap concentration c* is frequently estimated using the equation for a random coil in a good solvent [108]:

(12) $$c^{*}=\frac{3M_{n}}{4\pi N_{A}R_{g}^{3}}$$

where *N*_A_, *M*_n_ and *R_g_* are Avogadro’s number, the polymer number average molecular weight and the radius of gyration, respectively. At c*, a solution enters a semi-dilute regime with chains no longer separated. In another approach, the entanglement concentration, at which physical constraints (entanglements) lock the chains together, restricting their motion, may be applied to estimate the lower threshold for the critical gelation concentration [93].

For their random PNIPAM-butyl acrylate copolymers, Li et al. suggested that, upon heating, the hydrophobic microdomains were formed between the dehydrated NIPAM units and butyl groups. Such microdomains played the role of physical crosslinks and prevented the macromolecules from collapse and aggregation. The transparent gel phase represented an interconnected network with hydrophobic microdomains as network junctions. The formation of the opaque gel phase was attributed to micelle packing of the collapsed polymer chains. Lastly, the opaque hydrogels underwent macrophase separation upon heating, with water release and formation of the shrunk dehydrated gel phase.

In a series of physico-chemical and structural studies with the use of synchrotron small-angle/wide-angle X-ray scattering [93,96,97,98,99], Wang et al. thoroughly characterized the physical gel formation and evolution in atactic PNIPAM and built a model based on the “pearl-necklace” concept introduced by Okada and Tanaka in [39].

**Figure 9:**
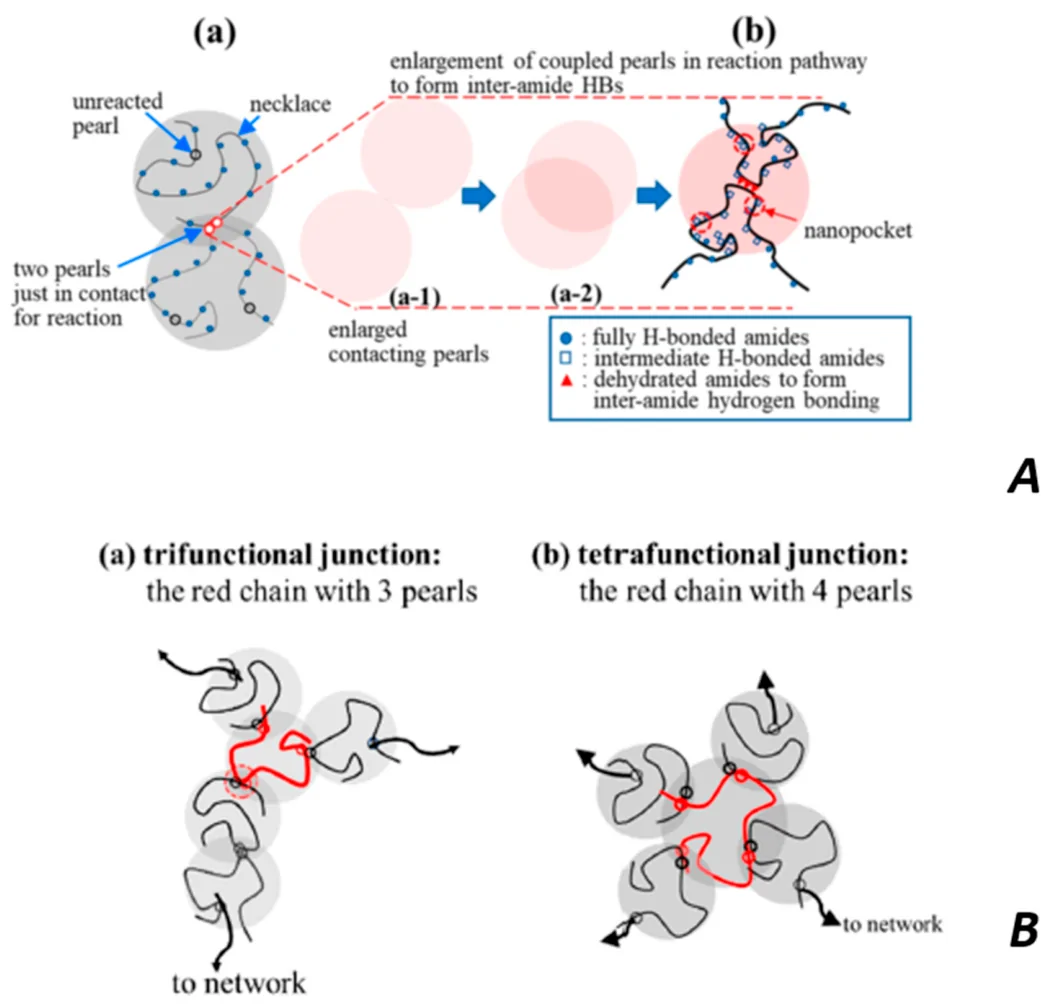
Model of physical gelation in PNIPAM based on the “pearl-necklace” concept [93,96]. (**A**) Proposed formation of a physical junction: (a) Two a-PNIPAM chains are connected via the hydrophobic interaction of the “pearls” in the overlapping region (red circles, reacted) to form “coupled pearls”; (b) enlargement of the “coupled pearls” to illustrate the collapsed chain segments to highlight the interchain association via inter-amide hydrogen bonding. (a-1) and (a-2) show the reaction pathway of the two contacting pearls that gradually overlap and eventually develop the inter-amide hydrogen bonds in (b). (**B**) Formation of trifunctional and tetrafunctional junctions. Reproduced from *Gels* under Creative Commons Attribution 4.0 International License.

According to the developed model, a pregel state is characterized by enhanced interchain associations. These pregel clusters are physically crosslinked to form a 3D network under conditions of concentration fluctuations upon further heating. If a PNIPAM chain is presented as a sequence of more hydrophobic compact “pearls” and hydrated “necklaces” [39], then the “pearls” are the most probable crosslinking sites for the formation of potential gel junctions. The contacts of two “pearls” are initiated by hydrophobic interactions and stabilized by van der Waals force and hydrogen bonds between the dehydrated amide groups. The two “pearls” contact, overlap, and finally coalesce to form a “coupled pearl” reducing the total hydrophobic surface exposed to water (Figure 9A).

The junction which brings two polymer chains together can extend them to form a network strand. A functionality of equal or higher than three is necessary for the formation of a 3D network. Thus, a chain must contain ≥3 “pearls” to form a gel network junction (Figure 9B). The main building blocks of a 3D network—strands and junctions—are formed by the interactions between “pearls” of different functionality. In a formed gel, these strands and junctions form a percolated macroscopic gel network. According to the SAXS/WAXD studies, the radius of gyration for a trifunctional junction in a gel was measured as 30−55 Å. The functionality of chains was found to increase with the temperature, resulting in the increase in a junction’s radius of gyration. The number of “pearls” per chain apparently grows with the chain length, thus more high-molecular PNIPAM forms hydrogels better, and the authors suggested that a certain minimum molecular weight of the polymer was needed for a chain to carry three or more crosslinking sites.

When the transparent gel of PNIPAM is heated to the temperature of the coil-to-globule transition, it transforms into the turbid gel. According to [98], in the process of spinodal decomposition the 3D network of a transparent gel undergoes phase separation with the formation of a sponge-like bicontinuous structure, which consists of a percolated domain network phase (enriched with the polymer) and a matrix phase (enriched with water). The network elasticity may lead to the cessation of the network coarsening and the prevention of the macroscopic phase separation, providing the gel stability.

It should be noted that the suggested model based on the “pearl-necklace” concept cannot be used for all such systems, first, due to inherent limitations (the separation into hydrated and dehydrated regions is an approximation, and even hydrophobic fragments of PNIPAM carry water molecules), second, it has been developed for neat PNIPAM, while the presence of substituents in a PNIPAM chain may lead to different mechanisms of gelation.

Table 2 summarizes the key concepts of the polymer phase behavior that can be controlled by various parameters and used in the practical design of PNIPAM-based injectable hydrogels.

## 8. Conclusions and Future Prospects

PNIPAM-based thermoresponsive hydrogels belong to the class of “smart” materials capable of changing their degree of swelling, mechanical properties, and shape in response to thermal stimuli. PNIPAM and its derivatives are of primary interest among thermoresponsive polymers due to the temperatures of phase transition that can be tuned near the body temperature. Both physical and chemically crosslinked PNIPAM hydrogels have been widely studied. The former are held together by noncovalent interactions, and their thermogelation is reversible. The latter have a stable 3D network bridged by chemical crosslinks. PNIPAM hydrogels retain large amounts of water and can accommodate loading and transport of various molecules and cells.

The structure and thermoresponsive properties of PNIPAM-based polymers make them attractive for a variety of biomedical and related applications, especially controlled drug delivery, cancer therapy, tissue engineering, wound dressings, and biosensors/smart textiles. Special attention is paid to injectable PNIPAM-based compositions, since they can be injected as liquids and then gel in situ, enabling minimally invasive delivery of drugs or cells.

The phase behavior of PNIPAM-based polymers in hydrogels is a key parameter which determines their properties and applications. PNIPAM represents an LCST-type polymer with a characteristic U-shaped phase diagram in mixtures with water; thus, a PNIPAM–water binary system exists as a solution (sol) below the binodal and demixes when heated to temperatures above the binodal, forming either turbid sols or hydrogels, depending on the polymer structure and concentration. The coil-to-globule transition of PNIPAM is entropically driven, due to the release of a large amount of water from the hydration shells of macromolecules at the transition temperature. Gelation in semi-dilute solutions, leading to the formation of a turbid physical gel, is a sol–gel transition. In chemically crosslinked gels, it is a volume phase transition, leading to the collapse of the whole network with the gel shrinking and expelling water.

Thermodynamically, PNIPAM and its derivatives do not exhibit the classical Flory–Huggins behavior, their phase transition showing weak dependence on the molecular weight and concentration, leading to what is described as Type II critical behavior. Physical gelation in PNIPAM solutions is caused by chain entanglement and binding between chains above the phase transition temperature, with the exact mechanism depending on the polymer architecture. Overall, the thermoresponsive behavior of PNIPAM and its derivatives is governed by the hydrophobic interactions, hydrogen bonding, and the rearrangement of hydration shells.

The presence of salts has been shown to strongly affect PNIPAM phase transition, following the Hofmeister series: kosmotropic ions disrupt the hydrogen bonds between the polymer macromolecules and molecules of water and promote the hydrophobic interaction, thus lowering the transition temperature (a “salting-out” effect); chaotropic ions tend to stabilize hydration and can raise the transition temperature (a “salting-in” effect). The mechanisms of their action include competition of ions with PNIPAM for water and disruption of amide hydration, increasing the surface tension of the cavity around the hydrophobic groups of the polymer, and direct ion binding to amide groups. The salt effect on the PNIPAM gelation and formed gel’s properties is an attractive way to tune the hydrogel behavior without the use of chemical modifiers or additional processing, e.g., in 3D bioprinting.

Pressure (mechanical stress) is another factor that strongly influences the phase behavior of PNIPAM-based systems. Increasing pressure impacts the state of the polymer’s hydration shells: the water structures become more ordered, and the hydration of hydrophobic groups is increased, thus favoring the coil state. The pressure–temperature phase diagram has a bell-like shape: at low pressure, the cloud point rises with pressure, reaches a maximum around a moderate pressure, and then decreases at higher pressure. In crosslinked hydrogels, pressure and mechanical constraints can shift or even suppress the phase transition. The load and constraints can also lead to the prolonged coexistence of both swollen and collapsed phases.

The future studies on the phase behavior of PNIPAM-based injectable hydrogels will be directed at the precise tuning of the gelation temperature, according to the specific biomedical needs, the development of compositions with better injectability and self-healing after extrusion, reduced burst release, and more controlled, sustained drug delivery. Development of multi-stimuli systems, exploiting other stimuli, besides the temperature, to induce the phase transitions, is in demand, along with applications of various factors which can influence the phase transitions. A rapidly developing field is the creation of multifunctional systems, in which the polymer phase transition is used for several purposes simultaneously, such as drug delivery and sensing.

Formation of hydrogels as a result of the coil-to-globule transition in semi-dilute solutions of PNIPAM-based polymers gives rise to the conventional thinking in the design of injectable hydrogels that gelation and coil-to-globule transition are concurrent processes. While several studies do provide direct evidence that the coil-to-globule transition and the sol–gel transition coincide for specific PNIPAM-based systems, in many other studies, this conclusion is inferred rather than experimentally proven. However, gelation in PNIPAM-based polymers may be a separate process from the coil-to-globule transition, producing a three-branched phase diagram. In this scenario, upon heating a polymer solution, first a transparent gel forms from a sol below the coil-to-globule transition temperature; the transparent gel undergoes a volume phase transition to a turbid gel upon reaching the coil-to-globule transition temperature.

The possibility of a phase diagram more complex than the common U-shaped phase diagram should be taken into account in the creation of injectable thermoresponsive hydrogels. Gelation in such systems takes place at a lower temperature than the LCST. On the one hand, it is a favorable situation for gelation inside the body and expands the temperature range of the polymer matrix workability. On the other hand, if the gelation temperature is rather low, it may need the injected solution to be refrigerated, since gel formation inside a syringe should be avoided. The pronounced dependence of the gel temperature on the polymer concentration may be considered a way of control over these properties.

Decoupled gelation and coil-to-globule transition may become advantageous for the design of injectable pharmaceutical formulations. The gel network formation follows the injection; however, no immediate water expulsion takes place, and the hydrogel remains transparent. The injectability window is broadened in this case, and burst release of a drug is eliminated, since no chain collapse occurs during the gelation. The latter gel property also ensures the gel stability over time and excludes the possibility of syneresis, probable at temperatures above that of the coil-to-globule transition. The hydrogel transparency creates a possibility of drug release monitoring using optical studies, as well as of local diagnostics based on optical measurements.

To date, there have been almost no studies on the use of such polymers as candidates for injectable gels. Tang et al. [102] studied the sustained release of methylene blue from an opaque gel of a diblock copolymer of PNIPAM, containing a glucose-bearing comonomer, which had both transparent and opaque gel regions. However, nothing is known so far about drug diffusion from transparent gels, as well as about the influence of the gel–gel transition on the potential injectable hydrogel drug- or cell-bearing properties. Apparently, diffusion of various substances in these gels is closely related to their inner structure and hydration; thus, such studies are warranted in the future. A separate problem to be studied concerns the influence of the Hofmeister series on both the gelation and coil-to-globule transition in such hydrogels which appears crucial for their performance.

In general, the conventional approach to injectable hydrogel design with the consideration of gelation and the coil-to-globule transition as one and the same process may not be correct for some PNIPAM copolymers and even for certain types of neat PNIPAM. Fundamentally, the complete phase diagram obtained with more than one technique is important, since it reveals the existence of multiple phases and a coil-to-globule phase transition which is no longer a sol–gel transition, but a volume phase transition from a transparent to an opaque gel. Practically, the premature gelation narrows the range of the solution injectability, the effect becoming more pronounced with the polymer concentration.

**Figure 10:**
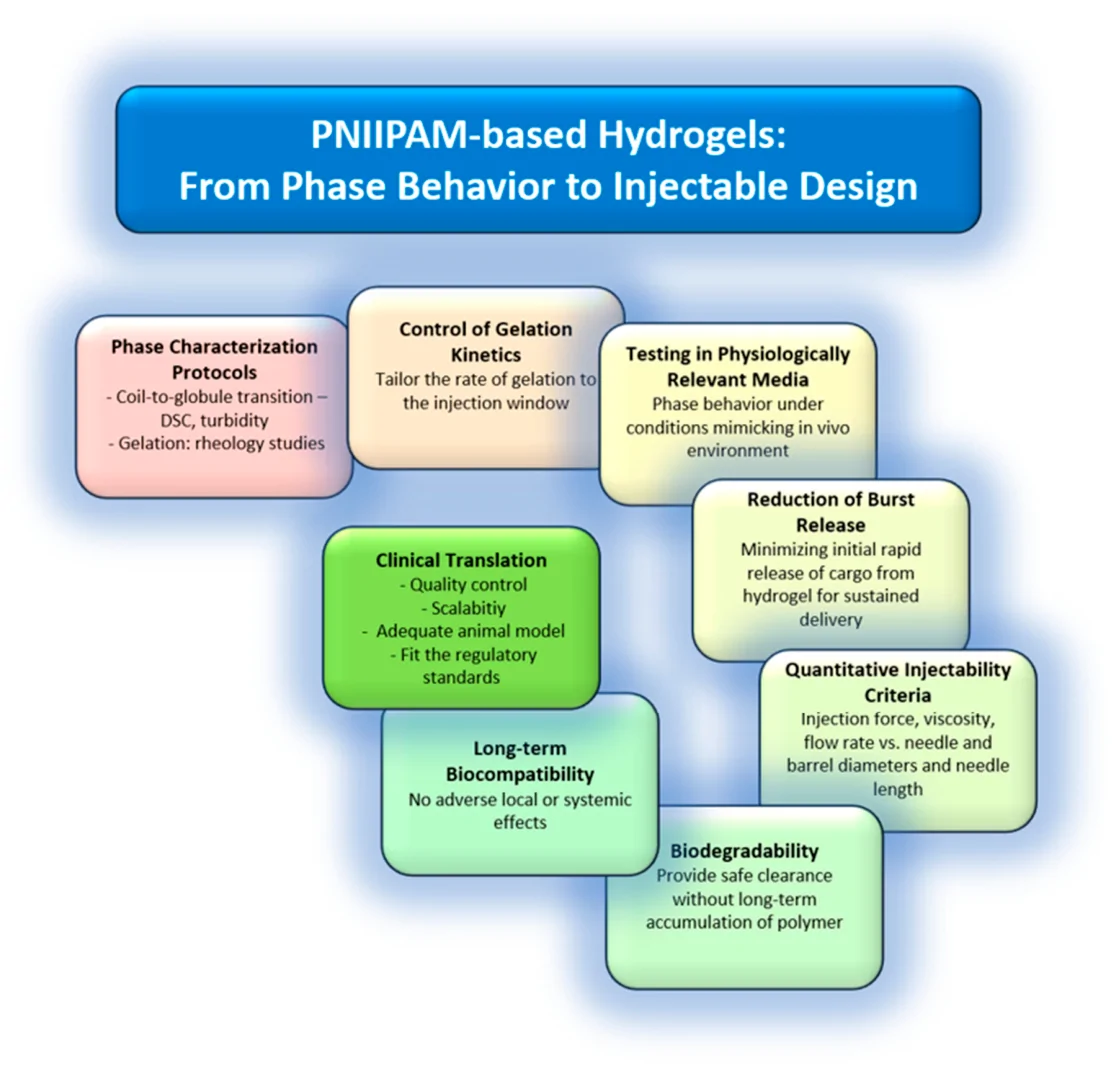
Pathway of PNIPAM-based injectable hydrogel design from laboratory research to clinical translation.

In conclusion, the thermoresponsive hydrogel design for biomedical applications should include sequential steps from thoroughly characterizing the phase behavior of a PNIPAM-based composition, verifying its consistency in physiologically relevant media, matching the kinetics of gelation and injectability requirements, to preventing burst release, providing the biodegradability and long-term biocompatibility, and achieving final clinical translation, involving the proper animal models, quality control, scalability, and compliance with the regulatory standards (Figure 10).

## Figures and Tables

**Table 1:** Critical temperatures used in the description of the PNIPAM thermal phase behavior.

	LCST	Cloud Point	VPTT
Definition	The lowest point of the binodal (demixing curve)—a purely thermodynamic term	The lowest point of an experimental cloud point curve	The lowest point of the demixing curve—used interchangeably as both a thermodynamic and experimental term
PNIPAM state below the critical point	Uncrosslinked polymer in a solution	Uncrosslinked polymer in a solution	Swollen crosslinked polymer (a gel or a microgel)
How the phase transition proceeds	Coil-to-globule transition in each macromolecule in a solution	Coil-to-globule transition in each macromolecule, leading to the formation of mesoglobules scattering light	Coil-to-globule transition leading to the swelling/deswelling of the entire network
Basic experimental techniques for the study	Strictly speaking, no experimental technique is capable of correctly measuring the LCST, since all the existing techniques need a certain amount of the formed new phase to detect the phase transition	Turbidimetry, DSC	Equilibrium swelling ratio studies, DLS for microgels

**Table 2:** Parameters affecting the phase behavior of PNIPAM-based polymers and their implications in the injectable hydrogel design.

Design Parameter	Effect on the Phase Behavior	Practical Implication for Injectable Hydrogel Design
Molecular architecture	Effect on the temperature of the coil-to-globule transition: hydrophobic units decrease the LCST, hydrophilic units increase the LCST. Different mechanisms of gelation depending on the chain structure (linear, block, graft, ABA/BAB-type, etc.): chain entanglement, micellar packing, intermicellar bridging, flower-like networks, etc.	Polymers with hydrophobic units form solutions which are favorable to gel inside the body, while hydrophilic units may hinder gel formation inside the body. Architectures prone to form micelles, such as block-type, are considered easier to form physical gels.
Molecular weight	The coil-to-globule transition demonstrates only weak dependence on the molecular weight. However, longer chains are inclined to greater intermolecular association and entanglement and thus favor gelation.	The choice of the molecular weight should be based on the balance between expanding the concentration region of gelation and increasing the solution viscosity leading to the impaired injectability.
Crosslinking density	Increasing the crosslinking density decreases the VPTT, broadens the region of the transition and decreases the volume change during the transition.	The choice of the crosslinking density should take into account the width of the transition, the required degree of deswelling during the transition and the mechanical strength of the resultant hydrogel after the injection.
Polymer concentration	The conventional phase diagram of PNIPAM is “flat”, i.e., the temperature of the coil-to-globule transition only weakly depends on the polymer concentration. However, a sufficiently high concentration is needed for gelation. In a three-branched phase diagram, the gelation temperature drops with the polymer concentration.	The concentration selection should be balanced between exceeding the critical gelation concentration and preserving the injectability. Shear-thinning behavior is also favorable for injectable hydrogels.
Salt composition and concentration	Kosmotropic salts decrease the coil-to-globule transition temperature, frequently approximately linearly with concentration. Chaotropic ions’ effect is more complex.	The hydrogels should be studied not only in pure water but also in the working medium. Adding salts can also be used to tune the temperature of gelation.
Injection pressure	The pressure–temperature phase diagram has a bell-like coexistence curve: transition temperature initially increases with pressure, reaches a maximum and subsequently decreases. Increasing pressure also broadens the transition.	In the case of viscous fluids, the pressures during the injection might be significant. Ideally, the pressure effect should not be neglected in the injectable hydrogel design
Shear stress during injection	The flow-induced shear can transform globules into the extended state even above the transition temperature.	The created hydrogel should be able to rapidly recover its structure after the injection
Mechanical constraints following the injection	The presence of mechanical constraints may influence the volume change, lead to the coexistence of swollen and collapsed domains, and even suppress the transition.	Gelation in a freely positioned sample may differ from that of the implanted composition. Additional studies under constrained conditions may be needed.
Phase diagram type	In the case of a conventional U-shaped phase diagram, gelation coincides with the coil-to-globule transition. For a three-branched diagram, first a transparent gel may form, which subsequently undergoes a volume phase transition to an opaque gel. More phases and more complex phase behavior are also possible.	It is best to use more than one technique to probe the coil-to-globule transition and gelation. Gelation in such systems takes place at a lower temperature than the LCST. On the one hand, it expands the temperature range of the polymer matrix workability. On the other hand, it may need the injected solution to be refrigerated, to avoid gel formation inside a syringe.

## Data Availability

No new data were created or analyzed in this study. Data sharing is not applicable to this article.

## Abbreviations

The following abbreviations are used in this manuscript:

PNIPAM	Poly(N-isopropylacrylamide)
LCST	lower critical solution temperature
UCST	upper critical solution temperature
VPTT	volume phase transition temperature
MD	molecular dynamics
FTIR	Fourier-transform infrared
DSC	Differential scanning calorimetry
BTA	Benzene-1,3,5-tricarboxamide
HEMA	2-Hydroxyethyl methacrylate
MAPLA	Methacrylate-polylactide
PDMA	Poly(N,N-dimethylacrylamide)
PVP	Polyvinylpyrrolidone
PEG	Polyethylene glycol
RAFT	Reversible Addition-Fragmentation Chain-Transfer
SAXS	Small-angle X-ray scattering
WAXD	Wide-angle X-ray diffraction
